# Sensation weighting in duration discrimination: A univariate, multivariate, and varied-design study of presentation-order effects

**DOI:** 10.3758/s13414-020-01999-z

**Published:** 2020-04-27

**Authors:** Åke Hellström, Geoffrey R. Patching, Thomas H. Rammsayer

**Affiliations:** 1 Department of Psychology, Stockholm University, SE-106 91 Stockholm, Sweden; 2 Lund University, Lund, Sweden; 3 University of Bern, Bern, Switzerland

**Keywords:** Duration discrimination, Presentation-order effect, Time-order error, Type B effect, Sensation weighting

## Abstract

Stimulus discriminability is often assessed by comparisons of two successive stimuli: a fixed standard (St) and a varied comparison stimulus (Co). Hellström’s sensation weighting (SW) model describes the subjective difference between St and Co as a difference between two weighted compounds, each comprising a stimulus and its internal reference level (ReL). The presentation order of St and Co has two important effects: Relative overestimation of one stimulus is caused by perceptual time-order errors (TOEs), as well as by judgment biases. Also, sensitivity to changes in Co tends to differ between orders StCo and CoSt: the Type B effect. In three duration discrimination experiments, difference limens (DLs) were estimated by an adaptive staircase method. The SW model was adapted for modeling of DLs generated with this method. In Experiments 1 and 2, St durations were 100, 215, 464, and 1,000 ms in separate blocks. TOEs and Type B effects were assessed with univariate and multivariate analyses, and were well accounted for by the SW model, suggesting that the two effects are closely related, as this model predicts. With short St durations, lower DLs were found with the order CoSt than with StCo, challenging alternative models. In Experiment 3, St durations of 100 and 215 ms, or 464 and 1,000 ms, were intermixed within a block. From the SW model this was predicted to shift the ReL for the first-presented interval, thereby also shifting the TOE. This prediction was confirmed, strengthening the SW model’s account of the comparison of stimulus magnitudes.

Participants in many psychological experiments have to compare the magnitudes of two stimuli. The outcome of such comparisons is not always as “common sense” would expect, which is still not fully explained. This is the point of departure of this study.

It is often assumed that comparative judgment is determined only by the difference between the stimuli’s magnitudes, as experienced one by one. According to this *simple difference model* of comparison (Thurstone, 1927a, 1927b), no systematic underestimation or overestimation of one stimulus relative to the other should occur, regardless of the order in which they are presented. Nevertheless, such effects do occur: Often, when two physically equal stimuli are compared, one of them tends to be judged as being greater (e.g., heavier or of longer duration) than the other. This kind of effect was first noted by the founder of psychophysics, Gustav Fechner (1860), who named it the time-order error (TOE). When the first stimulus is overestimated relative to the second stimulus, the TOE is positive, and in the opposite case, negative.

The Fechnerian TOEs have been the subject of much research throughout the years (see Hellström, 1985, for a review), and several explanations have been given. Most of these have assumed that the TOE is a perceptual/cognitive phenomenon. Yet, during the era of S. S. Stevens’s “new psychophysics,” it became an established “truth” that the TOE was due to a methodological flaw (Stevens, 1957) or to some form of judgment bias (Allan, 1977; Allan & Kristofferson, 1974; Engen, 1971; Luce & Galanter, 1963; Restle, 1961). However, Jamieson and Petrusic (1975) and Hellström (1977) varied the response format in TOE experiments and concluded from their results that a bias-based explanation could not hold: The TOE proved virtually insensitive to the response format—for instance, judging the second stimulus as less or greater than the first, or the first as less or greater than the second. Whereas Ulrich and Vorberg (2009) as well as Alcalá-Quintana and García-Pérez (2011) and García-Pérez and Alcalá-Quintana (2017, 2019) have maintained that judgment bias is the major determining factor of the TOE, most contemporary researchers emphasize perceptual-cognitive mechanisms (e.g., Bausenhart, Dyjas, & Ulrich, 2015; Hellström & Rammsayer, 2015; Patching, Englund, & Hellström, 2012; Preuschhof, Schubert, Villringer, & Heekeren, 2010; Raviv, Ahissar, & Loewenstein, 2012; van den Berg, Lindskog, Poom, & Winman, 2017). Nonetheless, stimulus comparison, like human judgment in general, cannot be expected to be free from bias, and this fact has to be taken into account. The most likely kind of bias in stimulus comparison seems to be “indecision bias” (García-Pérez & Alcalá-Quintana, 2017, 2019): When the participant compares two stimuli and must select one as being the greater, they have to guess when uncertain.

## Measurement of difference limens

Studies of the comparison of stimuli are often performed in order to measure discriminability, which is usually conceived in terms of a *difference limen* (DL; also, *just noticeable difference*). In typical experimental designs, based on the constant method (Guilford, 1954), a standard stimulus (St) and a comparison stimulus (Co) are presented in succession, St being held at a constant magnitude, and Co varying from trial to trial. Two so-called limens (thresholds) can then be determined: the upper limen (the value of Co that evokes 75% judgments of Co > St) and the lower one (the value of Co that evokes 75% judgments of Co < St). Both of the limens are affected when there is a TOE, so the DL is usually taken as half the difference between the upper and the lower limen (e.g., Luce & Galanter, 1963).

One problem with the DL is that its size has been found to depend on the presentation order of St and Co—that is, on whether the changes to be detected are in the first stimulus or the second one. Holding the first stimulus constant and varying the second one (order StCo) has an impact on the proportion of judgments of “second greater” that is often found to differ from what is obtained in the reverse procedure (order CoSt). Thereby, the two DLs will differ. This is called the Type B effect (Bausenhart et al., 2015; Ulrich & Vorberg, 2009), or standard position effect (SPE; Hellström & Rammsayer, 2015; Rammsayer & Wittkowski, 1990). In terms of DLs, the Type B effect can be defined as the difference DL_StCo_ − DL_CoSt_. Most often, the DL has been found to be smaller with the presentation order StCo than with CoSt, so that there is a negative Type B effect (Ellinghaus, Ulrich, & Bausenhart, 2018).

The TOE (also called the Type A effect) and the Type B effect make accurate determination of stimulus discriminability a methodological challenge that has been largely neglected, but it is a challenge that needs to be addressed. For instance, adequate assessment of duration discrimination is important in research on the neuropsychological basis of time perception (Rammsayer, 2008). To take account of the presentation-order effects, the simple difference model has to be replaced by a better one. This is also required for a deeper understanding of what goes on in our minds when we carry out the experimental—and also everyday—task of comparing two successive stimulus magnitudes.

## Modeling successive stimulus comparison

### Michels–Helson (MH) model

Michels and Helson (1954; also in Helson, 1964, Ch. 4) studied comparison of the magnitudes of two successive stimuli on a difference rating scale. They found, besides the TOE, that the scaled difference between the two stimuli was determined to a greater extent by the second-presented stimulus than by the first-presented one. The MH model states that the second-presented stimulus in the pair is not compared directly to the first-presented one, but to a weighted compound of the first-presented stimulus and the series adaptation level (AL). The latter is, in turn, a weighted geometric mean of previously experienced stimuli with weights according to their degree of recency—termed by Helson (1964) as series, background, and residual stimuli. Hence, *d*_12_^*^ = *u* {[*s* · ψ_1_ + (1 − *s*) ψ_*a*_] – ψ_2_}, where *d*_12_^*^ is the scaled stimulus difference, *u* is a scale factor, ψ_1_ and ψ_2_ are the subjective stimulus magnitudes, ψ_*a*_ is the subjective magnitude corresponding to the series AL, and *s* is the stimulus weight.

### Internal reference (IR) model

This model (Dyjas, Bausenhart, & Ulrich, 2012) bears similarity to the MH model. The second stimulus in a pair is not compared with the first stimulus, but to an IR. This IR is updated in a dynamic process, where the IR in the current trial is a weighted mean of the magnitudes of the first stimulus in the current pair (weight *g*; 0 < *g* < 1) and the IR in the previous trial (weight 1 − *g*): *d*_12_ = IR - ψ_2_ = [*g* · ψ_1_ + (1 − *g*) IR_*p*_] − ψ_2_, where ψ_1_ is the magnitude of the first stimulus of the current pair and IR_*p*_ is the previous IR. So, *g* thereby also becomes the impact weight of the first stimulus in its comparison with the second stimulus, which goes straight in with Weight 1. Therefore, in the constant method, the DL is predicted to be smaller when the second stimulus is varied (presentation order StCo) than with the order CoSt. This is, by definition, a negative Type B effect. The IR model predicts no TOE, which is because (unlike in the MH model) stimuli outside the series have no influence on the internal reference. As is noted by Dyjas and Ulrich (2014), “the [IR model] implicitly assumes that the Type B effect and the [TOE] are independent and that these effects reflect different underlying mechanisms” (p. 1139).

### Sensation-weighting (SW) model

For clarity, it is pertinent to revisit the origins of the SW model. Hellström (1979) carried out a loudness comparison experiment with 16 stimulus magnitude combinations in each of 16 combinations of stimulus duration and interstimulus interval. To describe the total set of data, a preliminary linear model was adopted which, in terms of subjective magnitudes, was *d*_12_^*^ = *B*_1*k*_· ψ_1_ – *B*_2*k*_· ψ_2_ + *C*_*k*_*,* where *d*_12_^*^ is the scaled subjective difference (calculated, for each stimulus combination [*k*], on group data for 12 participants, different for each condition), ψ_1_ and ψ_2_ are the magnitudes of the first and the second stimulus, *B*_1*k*_ and *B*_2*k*_ their regression coefficients, and *C*_*k*_ the intercept. This model was fitted to *d*_12_^*^ and to the physical stimulus magnitudes via a power function with a fitted exponent. Across conditions, *C*_*k*_ proved highly linearly dependent on *B*_1*k*_ and *B*_2*k*_. Using the best-fitting account of this dependence, *C*_*k*_ = *a*_2_*B*_2*k*_ – *a*_1_*B*_1*k*_ + *c*, the total number of fitted parameters in the model was reduced from 49 to 36, while preserving an excellent fit to the data (error variance 3.50% in the raw model and 4.94% in the accepted model). By analogy with the MH model, *a*_1_ and *a*_2_ were interpreted as *reference levels* (ReLs), ψ_*r*1_ and ψ_*r*2_, associated with the first and the second stimulus, respectively. *c* was interpreted as *u* (ψ_*r*1_ - ψ_*r*2_), where *u* is a scale factor. This resulted in the SW model, which can be written (Hellström, 1979; cf. Hellström, 1985, 2000, 2003; Hellström & Rammsayer, 2004, 2015):

1 $$ {d_{12}}^{\ast }=u\ \left\{\left[{s}_1\cdotp {\uppsi}_1+\left(1-{s}_1\right)\ {\uppsi}_{r1}\right]\hbox{--} \left[{s}_2\cdotp {\uppsi}_2+\left(1-{s}_2\right)\ {\uppsi}_{r2}\right]\right\}+b, $$

where *s*_1_ and *s*_2_ are the weighting coefficients of the stimuli, and ψ_*r*1_ and ψ_*r*2_ are their current ReLs. Judgment bias is represented by *b* (which was not included in the original version of the SW model).

The SW model is a natural generalization of the MH model, assuming that an adaptation-weighting mechanism operates on each of the compared stimuli, not only on the first one, so that the real comparison is not between the stimuli as such, but between two weighted compounds. Each of these compounds combines the subjective magnitudes of a stimulus and of its reference level (ReL). A ReL is conceptually similar to Helson’s (1964) adaptation level in being a product of the pooling of stimulus information from various sources. However, in the SW model the ReLs are not tied to Helson’s specifications of adaptation levels as weighted geometric means. The ReLs should usually be located near the center of the stimulus range, but have often been found to be slightly lower. ψ_*r*2_ may differ from ψ_*r*1_: Hellström (1979) found sound pressure levels of 67.38 dB and 68.20 dB corresponding to ψ_*r*1_ and ψ_*r*2_. Both of these are in the middle range of the stimulus magnitudes, but clearly below their mean dB value, 69.75 (the series AL value predicted by Helson’s theory). The difference between the two ReLs is likely to be due to the updating of ψ_*r*2_ with fresh magnitude information on the current ψ_1_.

Importantly, the formulation of the SW model in Equation 1 allows estimation of the scale factor *u*, and thereby of the “absolute” values of *s*_1_ and *s*_2_. These values, or their relation, are not subject to any formal restrictions. Although *s* values may usually be expected to stay between 0 and 1, indicating compromise or assimilation, Hellström (1979) obtained *s* values >1 in many stimulus conditions, implying negative weights for ψ_*r*1_ or ψ_*r*2_ − a contrast effect (Hellström, 1985).

The three models discussed are all built on the common, empirically well-grounded notion of stimulus comparison, as described by a linear model with different weights for the two stimuli. The SW model emerged as an extension of the MH model, generalized by assuming a weighting process for both of the stimuli, not just the first one. Like the MH model, the IR model corresponds to the SW model with *s*_2_ = 1 (cf. Bausenhart et al., 2015; Dyjas et al., 2012). However, unlike the MH model, the IR model recognizes no influence by stimuli external to the current experimental series (but see Bausenhart, Bratzke, & Ulrich, 2016). It may be noted that this limitation may be more realistic for studies where the standard stimulus is fixed within a block, as in the studies just cited, than for experiments where stimulus magnitudes show greater variation between trials (e.g., Hellström, 1979, 2003; Michels & Helson, 1954).

Unlike the other models discussed, the SW model places no restrictions on the values of *s*_1_ and *s*_2_. Thereby, it can account for such stimulus-condition dependent patterns of negative and positive TOEs and Type B effects as were found by Hellström (1979, 2003). The SW model has proved extremely useful for analysis and interpretation of the data in a number of later studies (e.g., Hellström & Cederström, 2014; Hellström & Rammsayer, 2015). In the present study, the SW model correctly predicts an experimental outcome.

### Explaining the TOE

In a common special case, ψ_*r*1_ can be assumed equal to ψ_*r*2_, and thereby both can be denoted by ψ_*r*_. In this case, letting ψ_1_ = ψ_2_ = ψ, Equation 1 becomes

2 $$ {d}_{12}=u\ \left({s}_2-{s}_1\right)\ \left({\uppsi}_r-\uppsi \right)+b $$

When two stimuli of equal magnitude are compared, a value of *d*_12_ ≠ 0 implies, by definition, a TOE. So, the SW model basically accounts for the TOE as being caused by the difference between stimulus weights, multiplied by the subjective difference between the ReL and the stimulus level, and, additionally, a judgment bias. With *s*_1_ < *s*_2_ and ψ_*r*_ below the mean level of ψ, this results in the common finding of a generally negative TOE. Also, in experiments with varying stimulus magnitude level, the TOE becomes negatively related to the current level, a relation that reverses in the rarer case of *s*_1_ > *s*_2_ (Hellström, 1979, 2003).

### Type B effect in the SW model

The SW model accounts for the Type B effect as being, like the TOE, a consequence of the differential weighting: The stimulus that is changed has an impact on the discriminative response in proportion to its weight (in presentation order StCo, *s*_2_, and in order CoSt, *s*_1_) and the DL is therefore inversely proportional to this weight.

Recently, Ellinghaus et al. (2018) surveyed the Type B effect across several stimulus continua, and maintained that when it is found, it is consistently negative, as predicted by the IR model. In contrast, results of Hellström and Rammsayer (2015) suggest that also positive Type B effects occur. Furthermore, results by Hellström (2003) and, in particular, Hellström (1979), obtained with methods that did not directly assess the DL, show equivalents (in terms of the SW model, *s*_1_ > *s*_2_) of large positive Type B effects for tonal loudness with brief stimuli and short interstimulus intervals. Verifying the results of Hellström and Rammsayer (2015) would therefore be of theoretical importance, as this would refute the MH and IR models, but would be consistent with the SW model. Such verification was attempted in the present study, for the case of duration discrimination, which is no exceptional case with regard to the phenomena just discussed (Eisler, Eisler, & Hellström, 2008; Ellinghaus et al., 2018).

### The present study

Hellström and Rammsayer (2004, 2015) used an adaptive staircase method to measure the DL for interval duration, with separate blocks for different stimulus presentation conditions. Experiment 2 in Hellström and Rammsayer (2015) employed filled auditory intervals, with St durations of 100, 215, 464, and 1,000 ms. In the present Experiment 1 we replicated this experiment with an improved procedure (see the Appendix). We also conducted two experiments with empty visual intervals (bounded by brief flashes): Experiment 2 (analogous to Experiment 1) and Experiment 3. In the two first experiments, we addressed perceptual-cognitive processes in duration discrimination, their expression as the TOE and the Type B effect, and their separation from judgment bias. In Experiment 3, we investigated whether, as is predicted by the SW model, the TOE can be shifted by manipulation of the ReLs. This attempted manipulation was done by using two St durations, instead of one as in Experiment 2, in each separate block of trials. The prediction was tested by comparing the results of Experiments 2 and 3.

## Experiments 1 and 2

In Experiments 1 and 2, duration discrimination was assessed with different presentation orders of standard (St) and comparison (Co) stimuli, and different St durations. DLs were measured using an adaptive two-alternative, forced-choice staircase method. Four interval durations were used in separate blocks. In Experiment 1, the intervals were filled auditory, and in Experiment 2, empty visual. These stimulus types were selected from those (also empty auditory and filled visual) used in Experiment 1 of Hellström and Rammsayer (2015) in order to confirm and further investigate the effect of stimulus duration on the size and direction of the Type B effect, which was found by Hellström and Rammsayer (Experiments 1 and 2) for these particular stimulus types.

### Method

#### Participants

Undergraduate psychology students at the University of Bern took part in the experiments. In Experiment 1, there were 57 females and eight males ranging in age from 19 to 48 years (*M* ± *SD* = 22.4 ± 4.3 years), and in Experiment 2, 44 females and 11 males, 19 through 29 years of age (21.3 ± 2.0 years). The participants received course credit. All of them were naïve about the purpose of the study and reported normal hearing and normal or corrected-to-normal vision. Because of the clear audibility or visibility of the stimuli, and the task being to compare the duration of the stimuli, not their magnitude, no further screening of hearing or vision was deemed necessary. All participants gave their written, informed consent.^1^

#### Apparatus and stimuli

Presentation of stimuli and recording of the participants’ responses were controlled by a computer program written in Turbo Pascal and an assembler-based timing routine. Timing accuracy of stimulus presentation was better than ±1 ms. Filled auditory stimuli (Experiment 1) were white-noise bursts presented binaurally through headphones (Sony CD 450) at an intensity of 66 dBA. Empty visual intervals (Experiment 2) were bounded by 3-ms flashes of a red light-emitting diode (LED; diameter 0.38°, viewing distance 60 cm, luminance 68 cd/m^2^) positioned at the eye level of the participant. The intensity of the LED was clearly above threshold, but not dazzling.

#### Procedure

The procedure was identical in Experiments 1 and 2. The participant was seated at a table with a keyboard and a computer monitor in a sound-attenuated and dimly lit room. To initiate the first trial, the participant pressed the space bar; the first stimulus interval was then presented after 900 ms, and then, after the 900-ms interstimulus interval, the second stimulus interval. Thereafter, the response was given by pressing one of two designated keys on the keyboard, labeled “first interval longer” and “second interval longer,” respectively. 2 Accuracy, not speed, was emphasized in the instructions. The next trial started 900 ms after the participant’s response. No correctness feedback was given.

##### Adaptive staircase method

A more detailed description of the psychophysical procedure is given in Rammsayer (2012). Participants compared the durations of two successive intervals, standard (St) and comparison (Co), using a two-alternative forced-choice response: “first interval longer” or “second interval longer.” On each trial of a series, the Co was increased or decreased in duration after having been judged as shorter or longer, respectively, than the St. A step that increased the absolute difference between Co and St was three times longer than a step that decreased this difference, which made performance settle at 75% responses of “first longer” or “second longer” (see Hellström & Rammsayer, 2015, for an explanation). Each participant took part in only one experiment, which was run in one experimental session consisting of eight blocks, with a 1-min break following each block. After six practice trials, the experimental session comprised four pairs of 64-trial blocks, each block pair using one St duration, with the order of the four St durations (100; 215; 464; and 1,000 ms) balanced across participants. Each block pair comprised one Hi-Co block, where Co was initially longer than St, and one Lo-Co block, where Co was initially shorter than St. For half of the participants, each block pair started with a Hi-Co block, and for the other half, with a Lo-Co block. Each block comprised two randomly interleaved 32-trial series, one series of pairs with an *Up* (U) profile, where the second interval was initially longer than the first, and one with a *Down* (D) profile, where the second interval was initially shorter than the first. So, with StCo and CoSt indicating the presentation order, the four series types were StCoU, StCoD, CoStU, and CoStD. Trials in a Hi-Co block were, equally often and in random order, from the StCoU and the CoStD series, and in a Lo-Co block, from the StCoD and the CoStU series.

When the St was 100 (215; 464; 1,000) ms, the initial duration of the Co in a series was 35 (70, 100, 500) ms below the St duration (in Lo-Co blocks) or above it (in Hi-Co blocks). The Co duration was then changed, using the weighted up–down method as described above, to estimate the upper or the lower DL (i.e., the duration difference for which 75% judgments of “first interval longer” or “second interval longer,” as pertinent, were obtained). In a Lo-Co (Hi-Co) block, the Co was increased (decreased) by 5 (9, 15, 100) ms after having been judged as shorter (longer) than the St, and decreased (increased) by 15 (27, 45, 300) ms after having been judged as longer (shorter) than the St. These steps were used for Trials 1–6; in Trials 7–32, the corresponding steps were 3 (6, 10, 25) and 9 (18, 30, 75) ms. See Table 6 in the Appendix for a summary of the procedure.

#### Measurement and modeling

##### Raw DLs.

In experiments where *d*_12_ is measured on each experimental trial (e.g., Hellström, 1979, 2003), fitting the SW model (Equation 1) to the data is quite straightforward. In contrast, what is measured in each condition of the present experiments is the value of Co that evokes 75% or 25% judgments of “first interval longer.” For each participant and each of the four conditions per St duration, the mean, across the last 20 trials, of the duration difference between the first and second presented stimulus (i.e., Co − St in CoSt series and St − Co in StCo series) was computed. From this we obtained the raw DL − rDL_D_ in D series and rDL_U_ in U series. At the rDL_D_ the *d*_12_ value corresponds to the 75th percentile, and at the rDL_U_ to the 25th percentile, in this participant’s distribution of *d*_12_ across trials. We denote these *d*_12_ values by *d*_12*x*_ and −*d*_12*x*_, respectively. The measured rDL values are, as is detailed in the text, subject to condition-specific effects, and they should not be taken as indices of discriminability.

##### **Modeling approach**

To model the participant’s comparison behavior, the SW model (Equation 1) was adapted to the particular type of experimental data obtained. Similar modeling was used in Hellström and Rammsayer (2004, 2015). The psychophysical function was assumed to be the identity function, ψ = ϕ, over the range of Co intervals for each St duration (no assumption was made concerning its shape across St durations). Also, *d*_12_ is specified in ϕ units, so that the scale factor *u* can be dropped. From Equation 1 we obtain

3 $$ {d}_{12}=\left[{s}_1{\upphi}_1+\left(1-{s}_1\right)\ {\upphi}_{r1}\right]-\left[{s}_2{\upphi}_2+\left(1-{s}_2\right)\ {\upphi}_{r2}\right]+b $$

For Experiments 1 and 2, the blocked design, with only one St duration per block, makes it reasonable to assume that the two ReLs are equal, ϕ_*r*1_ = ϕ_*r*2_ = ϕ_*r*_ (cf. Hellström, 2000), which yields the simpler expression

4 $$ {d}_{12}={s}_1{\upphi}_1-{s}_2{\upphi}_2+\left({s}_2-{s}_1\right)\ {\upphi}_r+b $$

The “noise” dispersion of *d*_12_ across trials, σ_*d*12_, may be termed the *comparatal dispersion* (Gulliksen, 1958), and we assume it to be proportional to the mean subjective stimulus magnitude (as per Ekman’s law; see Eisler et al., 2008). For simplicity, in the equations the physical magnitudes of the St and the Co, ϕ_*St*_ and ϕ_*Co*_, are abbreviated *S* and *C*. Our assumption ψ = ϕ then yields *d*_12*x*_ = *w*_*i*_ · *S* (as per Weber’s law in its simple form), where *w*_*i*_ is the participant-specific value of σ_*d*12_ / *S*, multiplied by 0.6745 (i.e., the standard normal deviate corresponding to the 75th percentile). We term *w* the *Weber constant*; *w* is not the same thing as a measured Weber fraction, but is assumed to underlie it. Judgment bias is likewise modeled as a participant-specific proportion of the St duration, *b*_*i*_ · *S*.

##### Weight ratio and Type B effect

As appropriate for each of the four series types (StCoU, StCoD, CoStU, CoStD), *S* and *C*, or *C* and *S*, were substituted in Equation 4 for ϕ_1_ and ϕ_2_, and the value of *d*_12_ was specified as either *d*_12*x*_ (in D series) or -*d*_12*x*_ (in U series). This resulted in Equations 14–17 (see the Appendix). From these equations we obtain, in terms of *Weber fractions* (WFs), where WF = DL/*S* and the WF for an individual series type is called a raw WF (rWF),

5 $$ {\mathrm{WF}}_{\mathrm{StCo}}=\left({\mathrm{rWF}}_{\mathrm{StCo}\mathrm{U}}+{\mathrm{rWF}}_{\mathrm{StCo}\mathrm{D}}\right)/2=w/{s}_2 $$

6 $$ {\mathrm{WF}}_{\mathrm{CoSt}}=\left({\mathrm{rWF}}_{\mathrm{CoSt}\mathrm{U}}+{\mathrm{rWF}}_{\mathrm{CoSt}\mathrm{D}}\right)/2=w/{s}_1 $$

Hence,

7 $$ {\mathrm{WF}}_{\mathrm{StCo}}/{\mathrm{WF}}_{\mathrm{CoSt}}={s}_1/{s}_2 $$

##### Estimation of model parameters from Weber fractions

For the mean WF across presentation orders, WF_M_, we have,

8 $$ {\mathrm{WF}}_{\mathrm{M}}=\frac{1}{2}\left({\mathrm{WF}}_{\mathrm{StCo}}+{\mathrm{WF}}_{\mathrm{CoSt}}\right)=\frac{1}{2}w\left({s}_1+{s}_2\right)/\left({s}_1{s}_2\right) $$

For *s*_1_ = *s*_2_ = *s*, WF_M_*= w/s*. From the data given in Table 7, in the Appendix, we obtained, with WFs estimated (by interpolation) at *s*_1_*/s*_2_ ≈ 1, rough estimates of *w/s*: 11.7% for Experiment 1 and 23.3% for Experiment 2.

The Type B effect is here defined as the Type B effect quotient (QTBE), the difference between the WFs in presentation orders StCo and CoSt as a fraction of WF_M_,

9 $$ \mathrm{QTBE}=\left({\mathrm{WF}}_{\mathrm{StCo}}-{\mathrm{WF}}_{\mathrm{CoSt}}\right)/{\mathrm{WF}}_{\mathrm{M}}=\left[w\ \left({s}_1-{s}_2\right)/\left({s}_1{s}_2\right)\right]/\left[\frac{1}{2}\ w\ \left({s}_1+{s}_2\right)/\left({s}_1{s}_2\right)\right]=2\left({s}_1-{s}_2\right)/\left({s}_1+{s}_2\right), $$

so that *s*_1_/*s*_2_ < 1 implies a negative, and *s*_1_/*s*_2_ > 1 a positive Type B effect.

##### Time-order errors (TOEs)

A positive (negative) TOE means that the first stimulus is overestimated (underestimated) relative to the second one. Thus, with a positive TOE, rDL_U_ (in U series) becomes larger than the corresponding rDL_D_ (in D series). One might attempt to estimate the TOE, for each presentation order (StCo or CoSt), as (rDL_U_ − rDL_D_)/2. However, it may theoretically be expected that the psychometric function, while symmetric on a logarithmic scale, is somewhat asymmetric on the linear duration scale, its slope being steeper at low than at high stimulus magnitudes (Eisler et al., 2008). Such an asymmetry would increase the DL in blocks of StCoU and CoStD (Hi-Co blocks; see the Appendix) as compared with blocks of StCoD and CoStU (Lo-Co blocks), and so bias the QTOE estimates (positively with the StCo order and negatively with the CoSt order). Such an effect is balanced out by defining the QTOE as its mean across presentation orders StCo and CoSt. Therefore, only this measure will be discussed in the following.

Adapting the SW model, as described in the Appendix, to fit the *S* and rDL values in each of the four series types yields Equations 17–20 (in the Appendix), which in turn yield Equations 18–21 that predict the rWFs from the SW model parameters. From these equations, the TOE quotient (QTOE), TOE/*S*, can be predicted as follows:

10 $$ \mathrm{QTOE}=\frac{1}{2}\ \left[\left({\mathrm{rWF}}_{\mathrm{StCoU}}-{\mathrm{rWF}}_{\mathrm{StCoD}}\right)/2+\left({\mathrm{rWF}}_{\mathrm{CoStU}}-{\mathrm{rWF}}_{\mathrm{CoStD}}\right)/2\right]=\frac{1}{2}\ \left\{\left[b+\left({s}_2-{s}_1\right)\ Q\right]/{s}_2+\left[b+\left({s}_2-{s}_1\right)\ Q\right]/{s}_1\right\}=\frac{1}{2}\ \left[b\ \left({s}_1+{s}_2\right)+Q\ \left({s_2}^2-{s_1}^2\right)\right]/{s}_1{s}_2, $$

where *Q* is the ReL distance quotient—that is, the relative distance of the ReL from the St: *Q* = (ϕ_*r*_ − *S*) / *S*.

##### Origin of QTOE

Equation 10 implies that QTOE depends on the weight difference as well as on the judgment bias, *b*. When the ReL is at a distance from the St, a QTOE arises from multiplication of *Q* by (*s*_2_^2^−*s*_1_^2^). With *Q* < 0, QTOE will be negatively related to (*s*_2_^2^−*s*_1_^2^), and thereby positively related to *s*_1_/*s*_2_.

Furthermore, it follows from the SW model that QTOE is closely related to QTBE. From Equations 9 and 10 we get

11 $$ \mathrm{QTOE}=\frac{1}{2}\ \left[b\ \left({s}_1+{s}_2\right)+Q\ \left({s_2}^2-{s_1}^2\right)\right]/{s}_1{s}_2=\frac{1}{2}\ b\ \left({s}_1+{s}_2\right)/{s}_1{s}_2-\frac{1}{2}\ Q\ \left({s}_1-{s}_2\right)\ \left({s}_1+{s}_2\right)/{s}_1{s}_2=\frac{1}{2}\ b\ \left({s}_1+{s}_2\right)/{s}_1{s}_2-Q\cdotp \mathrm{QTBE}\ \left[\frac{1}{4}\ {\left({s}_1+{s}_2\right)}^2/{s}_1{s}_2\right] $$

For *s*_1_= *s*_2_ = *s*, QTBE = 0, and QTOE = *b* / *s*. For a wide range of *s*_1_/*s*_2_ ratios, the factor 1/4 (*s*_1_+ *s*_2_)^2^ / *s*_1_*s*_2_ is close to 1, so that for moderate *b* values the slope of QTOE versus QTBE is predicted to be close to −*Q* (with QTOE and *Q* expressed in percentages).

### Results

All statistical analyses were conducted using IBM SPSS Statistics, Versions 25 and 26 for MacOS X.

#### Outlier exclusion

An initial screening for multivariate outliers (i.e., unusually deviating data patterns) was conducted, using the procedure described in Tabachnick and Fidell (2007, p. 74). Each participant’s squared Mahalanobis distance (based on the 16 rDLs) was tested against the χ^2^ distribution with *df* = 16 (matching the number of variables). Because of the limited number of participants in each experiment, failing to exclude a multivariate outlier might incur misleading results. Therefore, a criterion of *p* < .025 was used, instead of *p* < .001 as recommended by Tabachnick and Fidell. The test resulted in exclusion of the data from four participants in Experiment 1 and five participants in Experiment 2. Their exclusion was further justified by their squared Mahalanobis distances deviating clearly from the straight line in “Q–Q” plots of their quantiles against those of the χ^2^(16) distribution (cf. Garrett, 1989). Consequently, the analyses were based on *n* = 61 in Experiment 1, and *n* = 50 in Experiment 2.

##### Weber fractions

**Fig. 1:**
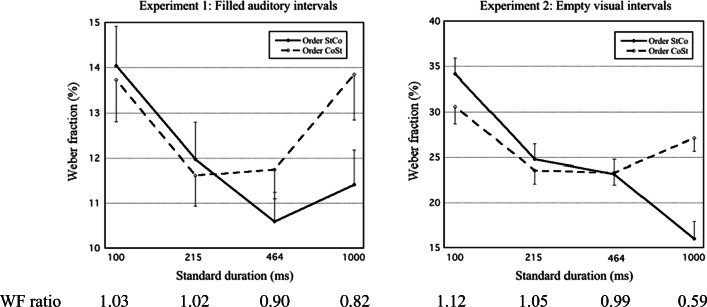
For Experiments 1 and 2, mean Weber fractions for presentation orders StCo and CoSt are plotted against standard (St) duration (logarithmic time scale). Error bars show the standard error of the mean (for clarity, drawn as one sided). Below the graph, the WF ratio WF_StCo_/WF_CoSt_ (which estimates *s*_1_/*s*_2_) is given for each St duration

For each experiment, descriptive statistics of rWF are given in Table 7, in the Appendix, for each of the four series types, as well as mean WFs for each St duration and across St durations. Nonpositive rWF values were observed in 7.1% and 4.6% of the cases in Experiments 1 and 2, respectively. For each experiment and St duration, the mean (*M*) and standard error of the mean (*SEM*) of the WF for each presentation order are shown in Fig. 1, as well as the estimate of WF_StCo_/WF_CoSt_ (indicating *s*_1_/*s*_2_).

For each experiment, the values of WF_StCo_ and WF_CoSt_ for each of the four St durations were submitted to a repeated-measures ANOVA, with St duration (100; 215; 464; 1,000 ms) and presentation order (StCo, CoSt) as within-participant factors. Here, as in all our ANOVAs, multivariate (Pillai) tests were used. The results are given in Table 1.

**Table 1.** ANOVA table for Weber fractions (WFs) from Experiments 1 and 2

	Experiment 1:Filled auditory intervals				Experiment 2:Empty visual intervals			
Effect	*F*	*df*	*p*	η_p_^2^	*F*	*df*	*p*	η_p_^2^
St duration	4.848	3, 58	**.004**	.200	17.415	3, 47	**< .001**	.526
Linear	2.271	1, 60	.137	.036	46.269	1, 49	**< .001**	.486
Quadratic	9.082	1, 60	**.004**	.131	14.626	1, 49	**< .001**	.230
Cubic	0.120	1, 60	.730	.002	3.423	1, 49	.070	.065
Order	3.278	1, 60	.075	.052	2.396	1, 49	.128	.047
Dur. × Order	2.414	3, 58	.076	.111	11.490	3, 47	**< .001**	.423
Linear	7.445	1, 60	**.008**	.110	29.752	1, 49	**< .001**	.378
Quadratic	0.666	1, 60	.418	.011	9.275	1, 49	**.004**	.159
Cubic	0.197	1, 60	.659	.003	2.631	1, 49	.111	.051
Type B effect St = 100 ms St = 215 ms St = 464 ms St = 1,000 ms	*t* 0.379 0.407 −1.501 −2.770	*df* 60 60 60 60	*p* − − .554 **.030**		*t* 2.089 0.724 −0.093 −5.536	*df* 49 49 49 49	*p* .168 − − **< .001**	

*Note.* Bonferroni-corrected *t-*test results for Weber fraction difference between presentation orders StCo and CoSt (i.e., Type B effect), are also given for each standard duration; *p* values indicating statistical significance (*p* < .05) are given in boldface

##### TOE Quotient (QTOE)

**Fig. 2:**
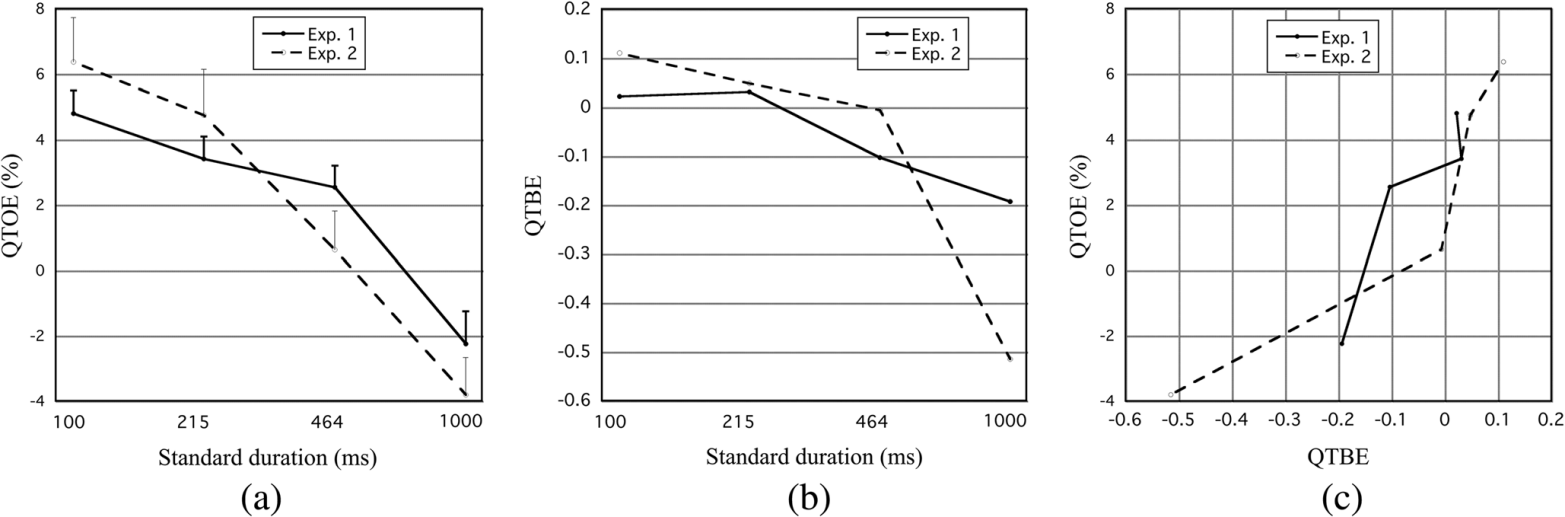
For Experiments 1 and 2, (**a**) TOE quotient (QTOE) is plotted against standard (St) duration (logarithmic time scale). Error bars show the standard error of the means (for clarity, drawn as one sided); (**b**) Type B effect quotient (QTBE; i.e., [WF_StCo_ − WF_CoSt_] / WF_M_) is plotted against standard (St) duration (logarithmic time scale); (**c**) QTOE is plotted against QTBE

Descriptive statistics of QTOE for each St duration are given in Table 7, in the Appendix. The means and their standard errors are shown in Fig. 2. For St durations that yielded values of *s*_1_*/s*_2_ near 1 (i.e., 215 and 464 ms) QTOE was positive, indicating *b* > 0—that is, a judgment bias in the direction of “first interval longer.” Using Equation 11, *b/s* was preliminarily and roughly estimated as the mean QTOE value for these durations, about +3.5% for both experiments.

For each experiment, the eight QTOE values were submitted to a repeated-measures ANOVA with St duration (100; 215; 464; 1,000 ms) and presentation order (StCo, CoSt) as within-participant factors. The results are given in Table 2.

**Table 2.** ANOVA table for time-order error quotients (QTOEs) from Experiments 1 and 2

	Experiment 1:Filled auditory intervals				Experiment 2:Empty visual intervals			
Effect	*F*	*df*	*p*	η_p_^2^	*F*	*df*	*p*	η_p_^2^
St duration Linear Quadratic Cubic	14.577 38.745 9.225 4.959	3, 58 1, 60 1, 60 1, 60	**< .001** **< .001** **.004** **.030**	.430 .392 .133 .076	8.907 27.528 2.051 0.378	3, 47 1, 49 1, 49 1, 49	**< .001** **<.001** .158 .542	.362 .360 .040 .008

*Note. p* values indicating statistical significance (*p* < .05) are given in boldface

#### Interpretation of univariate results

The SW model (Equation 1) describes the perceptual stimulus-comparison mechanism as being based on a comparison between two weighted compounds, each comprising a stimulus magnitude and a ReL. Accordingly, the model predicts that the weighting is reflected in Weber fractions as well as in TOEs.

##### Weber fractions

Equation 9 predicts that QTBE changes with the weighting balance (specifically, [*s*_1_ – *s*_2_] / [*s*_1_ + *s*_2_]) across St durations. In accordance with this, the ANOVA of WFs for Experiment 2 showed a significant St Duration × Order interaction, *p* = .003, to which the linear effect of St duration made the greatest contribution. Thus, the Type B effect—the effect of presentation order on the WF—was not constant, but changed with the St duration. However, in post hoc *t* tests the only clearly significant evidence for a nonzero Type B effect occurred for the 1,000-ms St duration, where the effect was negative (implying *s*_1_/*s*_2_ < 1).

For Experiment 1, the St Duration × Order interaction failed to reach statistical significance, *p* = .076. Still, one may note that the linear contribution of St duration to this interaction was significant, *p* = .008.

##### TOE quotients

Equation 10 implies that QTOE should be directly related to *Q* (*s*_2_^2^ − *s*_1_^2^) / (*s*_1_*s*_2_). Figure 2 gives some support to this, as it shows QTOE to be generally positively related to QTBE, and thereby to *s*_1_ − *s*_2_. This suggests that in each block *Q* < 0 (i.e., the ReL falls below the St). From the slopes of the linear regressions (QTOE vs. QTBE, group data) depicted in Fig. 2c, *Q* was estimated as −26.0% for Experiment 1 (*r* = .91) and −14.6% for Experiment 2 (*r* = .92). The *b* values were estimated as equal to the regression intercepts, +3.7% (Experiment 1) and +3.3% (Experiment 2).

The negative *Q* values are as could be expected from the results of Hellström and Rammsayer (2015). They are also in harmony with results for weight comparison with a single standard (Hellström, 2000). A parallel is the finding in temporal bisection experiments, where participants classify intervals as long or short, that the bisection (neutral) point is located below the arithmetic mean of the interval durations (Brown, McCormack, Smith, & Stewart, 2005; Wiener, Thompson, & Coslett, 2014). Similar findings were addressed by Helson (e.g., 1964) by specifying the adaptation level as a weighted geometric mean of the stimulus magnitudes.

##### Model fitting by NLR

For additional guidance regarding model parameters, Equations 18–21, in the Appendix, were used to fit the SW model, using the SPSS routine nonlinear regression (NLR). For each experiment, all the individual rWF estimates were entered together. *Q*, *w*, and *b* were assumed to be constant across conditions, and *s*_1_ and *s*_2_ to be condition specific. Only the value of *Q* could be uniquely estimated; *s*_1_, *s*_2_, *b*, and *w* were estimated relative to each other. Using the formula WF_M_ = *w/s* with the above WF_M_ estimates of 11.7% (Experiment 1) and 23.5% (Experiment 2), the values of *w* were fixed at 5.85% for Experiment 1 and at 11.75% for Experiment 2 to yield plausible average values for *s*_1_ and *s*_2_ of about 0.5 (cf. Hellström, 2003). The NLR results are given in Table 3. The model used in this analysis is obviously simplified, and *R*^2^ (corrected) is modest: .133 (Experiment 1) and .152 (Experiment 2), so the results should only be taken as guidance. Nevertheless, they generally confirm the preliminary results.

**Table 3.** Results from model fitting by SPSS NLR

St (ms)	Experiment 1:Filled auditory intervals			Experiment 2:Empty visual intervals		
	*s*_1_	*s*_2_	*s*_1_/*s*_2_	*s*_1_	*s*_2_	*s*_1_/*s*_2_
100	0.425 (0.022)	0.418 (0.022)	1.017	0.391 (0.020)	0.339 (0.015)	1.153
215	0.495 (0.029)	0.499 (0.027)	0.992	0.526 (0.034)	0.455 (0.025)	1.156
464	0.512 (0.031)	0.532 (0.030)	0.962	0.485 (0.030)	0.536 (0.033)	0.905
1,000	0.417 (0.025)	0.525 (0.039)	0.794	0.434 (0.026)	0.729 (0.070)	0.595

*Note*. Estimates (*SE*s in parentheses) of weights (*s*_1_ and *s*_2_), judgment bias (*b*, in %), and ReL distance quotient (*Q*). Except for *Q*, estimates are relative to fixed value of Weber constant (*w*, in %). Experiment 1: *w*_fixed_ = 5.85%; *b* = 1.83% (0.70); *Q* = −27.04% (13.32). Experiment 2: *w*_fixed_ = 11.75%; *b* = 1.47% (0.39); *Q* = −13.58% (3.51)

#### Multivariate approach: Principal component analyses of raw Weber fractions

Although the Type B effect clearly changed with St duration, unequivocal statistical evidence of its reversal (from negative to positive) for brief St durations was not obtained from our univariate analyses, as reported in Table 1. It also appears hazardous to build theoretical conclusions solely on measures built up by combinations of different forms of the rWF, each of which is highly variable across individuals.

However, this interindividual variability of the rWFs is a liability that can be turned into an asset: It carries information that is lost in univariate statistics. An attempt was therefore made to assess the parameters of the SW model by analyzing the *multivariate* variability of the rWFs.

##### Multivariate model

The multivariate model and its application to each of the series types is described in the Appendix. Equation 22, in the Appendix, corresponds to the basic model of principal component analysis (PCA), with components corresponding to *w* (Weber constant), *b* (judgment bias), and *Q* (relative distance of ReL from St). These components were therefore expected to emerge in a PCA of the rWFs for the 16 conditions (without rotation of extracted components). The eigenvalue of each component should then measure its contribution to the variability in rWFs. The calculated component scores for the *i*th participant should estimate this participant’s standardized values of *w*_*i*_, *b*_*i*_, and *Q*_*i*_, respectively. The three components’ loadings for the *k*th condition should estimate its values of ω_*k*_ (discrimination difficulty), β_*k*_ (bias expression), and δ_*k*_ (weight difference expression), respectively.

##### Analogy with ability testing

A useful analogy could be to think of each experiment as an ability-test battery, the *i*th participant’s characteristics (Weber constant, *w*_*i*_; judgment bias, *b*_*i*_; ReL distance quotient, *Q*_*i*_) being scores on three basic abilities, and the *k*th condition being one of 16 heterogeneous tests. Each test (i.e., condition) has loadings on *w*, *b*, as well as *Q*. As there is thus no “simple structure” that could be revealed by rotation, an unrotated PCA is appropriate. When the PCA is conducted on the “battery”—that is, the rWFs in the 16 conditions of the experiment—three components, corresponding to *w*, *b*, and *Q*, respectively, would then be expected to be extracted, in an order corresponding to their contribution to the total variance.

##### Principal component analyses (PCAs)

For each experiment, the 16 rWFs in the four series types (i.e., StCoU, StCoD, CoStU, and CoStD) for each of the four St durations (100; 215; 464; and 1,000 ms), were submitted to a PCA, using the FACTOR routine in SPSS. The Kaiser–Meyer–Olkin (KMO) measure of sampling adequacy^3^ (Kaiser, 1974) was .657 for Experiment 1 and .635 for Experiment 2. For each experiment, three components were extracted, with eigenvalues of 3.9 (explaining 24.4% of the variance), 3.1 (19.2%), and 1.6 (9.9%) for Experiment 1, and 4.4 (27.2%), 2.8 (17.7%) and 1.7 (10.4%) for Experiment 2.

##### Results of the PCAs

The unrotated component loadings are given in Table 8 in the Appendix. Scores of the three extracted components (*w*_*i*_, *b*_*i*_, *Q*_*i*_) were also computed for each participant. For an interpretation of the loadings, note that in Equations 18–21, in the Appendix, *w* always occurs as a positively signed term, whereas the *b* term is positively signed for Up (U) series, and negatively signed for Down (D) series.

For Experiment 1, the first component had (after reversal of loading signs) positive loadings for U series and negative loadings for D series, and individual component scores correlated highly with QTOE (see Fig. 5). It could thereby be identified as *b*, the loading for condition *k* indicating this condition’s bias expression, β_*k*_. The second component, whose scores correlated highly with WF_M_ and whose loadings (except one) were positive, could be identified as *w,* the loading for condition *k* indicating this condition’s discrimination difficulty, ω_*k*_.

For Experiment 2, the first component was identified as *w* (all loadings positive, highly correlated with WF_M_) and the second (after reversal of signs) as *b* (scores highly correlated with QTOE, loadings generally positive for U series and negative for D series). For each experiment, the third component was identified as *Q* (ReL distance quotient), its loading for condition *k* reflecting the weight difference, δ_*k*_, in this condition, that is, the multiplier of *Q*_*i*_ in determining the QTOE. The results are consistent with weight ratios *s*_1_/*s*_2_ > 1 for St durations of 100 and 215 ms, and *s*_1_/*s*_2_ < 1 for 464 and 1,000 ms (as was found from the analysis of WF_StCo_/WF_CoSt_ ratios) in combination with *Q* < 0 (i.e., the ReL being situated below the St) for each St duration.

In Table 8, in the Appendix, mean values of ω*,* β*,* and δ for each St duration are given, as estimated from the mean component loadings using Equation 22, in the Appendix. For Experiment 1, β (bias expression) was positive for each St duration, which indicates, in accordance with the estimated positive *b* value for *s*_1_/*s*_2_ = 1, a judgment bias that favors judgments of “first interval longer” for all St durations. For Experiment 2, such a bias was obtained for all St durations except 1,000 ms, where the bias was close to zero.

##### *Variance components in the comparison process*

As predicted by Equations 18–21, in the Appendix, the measured rWF is affected by the SW mechanism as well as by two participant-specific factors—namely, Weber constant (*w*) and judgment bias (*b*). The present experimental design made it possible to estimate, using PCA, the contributions of each of these factors to the total variance of the rWFs. As assessed by eigenvalues from PCAs of the rWFs, *w* and *b* dominated in this respect, leaving about 10% for the ReL distance quotient *Q*, the latter factor generating systematic TOEs by multiplication with the weight difference (*s*_2_*− s*_1_). This effect was limited by the blocked design, with the St duration fixed within each block, which minimized the possible asymmetry of *Q* as well as its interindividual variation. As is demonstrated in the next section, the role of *Q* in modulating the shift of QTOE with the St duration was still considerable, as was predicted from the SW model.

#### Relating PCA-estimated model parameters to univariate results: Comparison of univariate results from participants with low, medium, and high PCA component scores

**Fig. 3:**
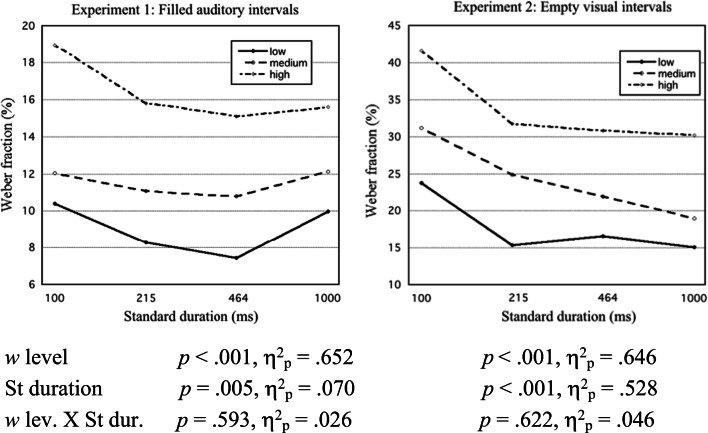
For Experiments 1 and 2, mean Weber fraction is plotted against standard (St) duration (logarithmic time scale) at low, medium, and high third score levels of *w* component. Included are ANOVA results for Weber fractions

**Fig. 4:**
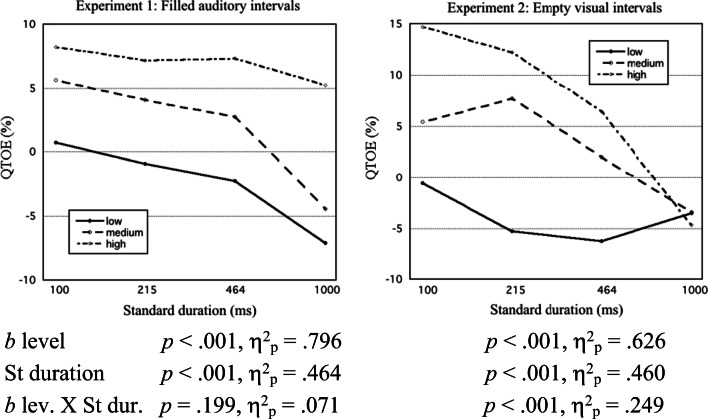
For Experiments 1 and 2, mean TOE quotient (QTOE) is plotted against standard (St) duration (logarithmic time scale) at low, medium, and high third score levels of *b* component. Included are ANOVA results for QTOEs

**Fig. 5:**
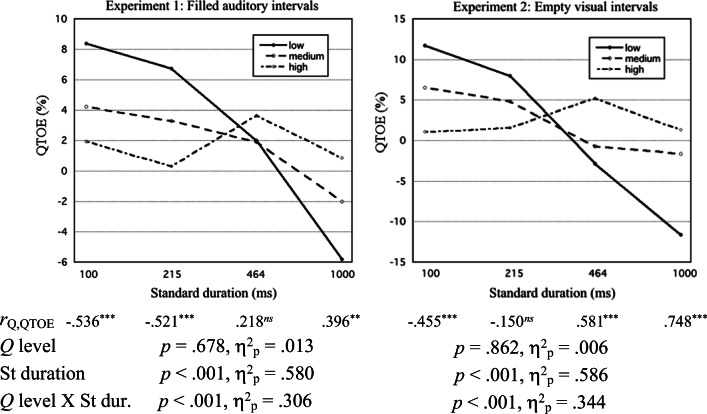
For Experiments 1 and 2, mean TOE quotient (QTOE) is plotted against standard (St) duration (logarithmic time scale) at low, medium, and high third score levels of *Q* component. Correlation between *Q* component and QTOE is given for each St duration (Bonferroni corrected: ^***^p < .001, ^**^p < .01, *ns* = not significant). Included are ANOVA results for QTOEs

For each of the three extracted components, the scores were partitioned at their low, medium, and high tertiles. Each of Figs. 3, 4, and 5 shows mean WF or QTOE for each partition of a component score, and is supplemented with ANOVA results.

##### Weber fractions (WFs)

Figure 3 shows, plotted against the St duration, the mean WF for participants with lowest, medium, and highest third levels of the *w* (Weber constant) component score. As expected, mean WFs increased with increasing *w* scores.

##### TOE quotients (QTOEs)

Figure 4 shows, in the same manner, the mean QTOE for participants with lowest, medium, and highest third levels of the *b* (judgment bias) component score. Mean QTOEs were directly related to *b* scores, except for Experiment 2 with *S* = 1,000 ms.

Finally, Fig. 5 shows the mean QTOE for participants with lowest, medium, and highest third levels of the *Q* (ReL distance quotient) component score. Correlations of the *Q* score with QTOE are also given for each St duration. According to the SW model, QTOE is proportional to the squared-weight difference (*s*_2_^2^ − *s*_1_^2^), multiplied by *Q*. As is shown in Fig. 5, and verified by the ANOVA results, scores of the *Q* component indeed modulated the slope of QTOE against St duration, and thereby against weight difference. This slope did not become positive even with the highest *Q* scores.

This suggests that most individual *Q* values stayed on the negative side. In the univariate analyses we found evidence (clearly significant only for Experiment 2) that the difference *s*_2_ − *s*_1_ was positive for *S* = 1,000 ms. This is confirmed by the significantly positive correlations between QTOE and *Q* component score for this St duration. Conversely, the significantly negative correlations for, in particular, St = 100 ms in both experiments indicate negative values of (*s*_2_ − *s*_1_). So, the univariate indications were confirmed: The weighting balance did reverse into *s*_1_/*s*_2_ > 1 (equivalent to a positive Type B effect) for brief St durations; significantly so for St = 100 ms (Experiments 1 and 2) and for St = 215 ms (Experiment 1).

#### Response times

Response times in Experiments 1 and 2 are reported and discussed in the Appendix.

## Discussion of Experiments 1 and 2

### Weighting change and its interpretation

The present results are generally consistent with those of Hellström and Rammsayer (2015). In particular, in both studies, the ratio *s*_1_/*s*_2_ tended to decrease with increasing stimulus duration. This parallels the decrease of *s*_1_/*s*_2_ with increasing interstimulus interval that generally occurs in TOE experiments (e.g., Hellström, 1979, 2003). The interval between the onsets of the first and the second stimulus increases with the interstimulus interval as well as with stimulus duration, so it seems likely that both of these temporal factors contribute to the change of the weighting balance.

This change, to the disadvantage of the first stimulus, has been proposed to reflect the tuning of a mechanism that increases discrimination sensitivity by optimal weighting-in of ReL magnitude information (Hellström, 1989; Patching et al., 2012; cf. Preuschhof et al., 2010). In particular, the weighting change is thought to reflect a transition, with longer interstimulus intervals and/or stimulus durations, from stimulus interference to memory loss.

Taking advantage of the interindividual variability provided the extra statistical power needed to confirm the reversal of the weighting pattern (i.e., yielding *s*_1_ > *s*_2_) with brief St durations. Similarly, in Hellström and Rammsayer (2004), for duration comparison of filled auditory intervals across interstimulus intervals of 100–2,700 ms, *s*_1_/*s*_2_ > 1 was generally found for St durations of 50 ms, and *s*_1_/*s*_2_ < 1 for 1,000 ms.

### Time order errors (TOEs)

Figures 3, 4 and 5 suggest that our univariate and multivariate analyses of the rWFs captured the essential factors in the build-up of the TOEs: sensation weighting and judgment bias. Importantly, positive as well as negative TOEs were shown to occur even with a blocked design, that is, in the absence of trial-to-trial variation of the St duration.

**Fig. 6:**
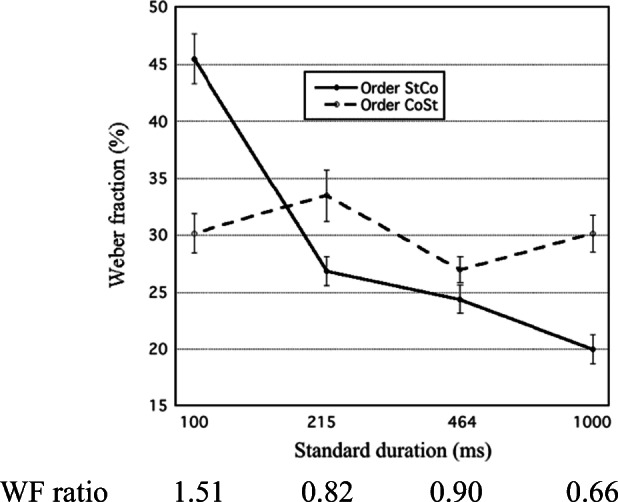
For Experiment 3 (empty visual intervals) mean Weber fraction, for presentation orders StCo and CoSt, is plotted against standard (St) duration (logarithmic time scale). Error bars show the standard error of the mean. Below the graph, the ratio WF_StCo_/WF_CoSt_ (which estimates *s*_1_/*s*_2_) is given for each St duration.

Judgment bias (*b*) contributes considerably to the interindividual variation of the TOE, but only moderately to its mean value across individuals. The bias and its interindividual variation are most easily understood as being due to individual guessing habits in cases of uncertainty (García-Pérez & Alcalá-Quintana, 2017). In Experiment 2, the impact of judgment bias vanished for the St duration of 1,000 ms. This may be due to participants using different guessing strategies for uncertain cases with the longest St duration than with shorter durations.

According to the present results, judgment bias does not account for the existence of the TOE or its variation across St durations and presentation orders. Instead, sensation weighting appears to be a major factor behind the TOE. In Experiment 3, this interpretation was put to a direct test.

## Experiment 3

### Background

In Experiments 1 and 2, one single St duration was used in each experimental block. This resulted, according to our findings, in values of *Q* (ReL distance quotient; i.e., relative dislocation of ϕ_*r*_ from the St duration) that were consistently negative.

### Manipulating the TOE

So far, only indirect evidence was obtained for the corollary of the SW model that *Q*, multiplied by the weight difference (*s*_2_ − *s*_1_), affects the subjective stimulus difference, and thereby determines the QTOE. So, in Experiment 3, using empty visual intervals like in Experiment 2, an attempt was made to manipulate *Q*, and thereby the QTOE.

### Double-standard design

A variation of the blocked experimental design, intermixing two St durations in the same block, offers an opportunity for an experimental test of this prediction. Thus, the procedure was modified so that in each block two St durations, short (100 and 215 ms) or long (464 and 1,000 ms), alternated randomly.

#### Modeling for the double-standard design

For this type of design, it cannot be assumed that the two ReLs are equal (i.e., that ϕ_*r*1_ = ϕ_*r*2_). We therefore return to the basic version of the SW model, in the form of Equation 3. This results in equations for the rWF in the four series types. These equations (24–27) are given in the Appendix. From those equations we obtain

12 $$ \mathrm{QTOE}=\left[\left({\mathrm{rWF}}_{\mathrm{StCoU}}-{\mathrm{rWF}}_{\mathrm{StCoD}}\right)+\left({\mathrm{rWF}}_{\mathrm{CoStU}}-{\mathrm{rWF}}_{\mathrm{CoStD}}\right)\right]/4=\left[\left(1-{s}_1\right)\ {Q}_1-\left(1-{s}_2\right)\ {Q}_2+b\right]\ \left(1/{s}_1+1/{s}_2\right)/2 $$

It follows that if, under otherwise unchanged conditions, *Q*_1_ or *Q*_2_ is manipulated, this will shift QTOE, in a manner determined by the values of (1 − *s*_1_) or (1 − *s*_2_), respectively. In Experiment 3, such manipulation was attempted by including pairs with two different St durations in random order (100 and 215 ms, or 464 and 1,000 ms) in the same experimental block.

In the double-standard design, when awaiting the first interval in the pair, participants cannot prepare for a particular approximate interval duration, and adjust ϕ_*r*1_ accordingly. Instead, they are expected to use a default value of ϕ_*r*1_. Having perceived the first-presented interval, the participant will then adjust ϕ_*r*2_ in the direction of this interval. It is here assumed that ϕ_*r*1_ will be close to the geometric mean of the two St durations in the block (cf. Helson, 1964), and that, in logarithmic measure, ϕ_*r*2_ will be adjusted from this in the direction of the first stimulus in the current pair by 20% of the distance (by analogy with results in Hellström, 1979, 2003). Expressed in terms of weighted geometric means, we have, on average, ϕ_*r*1_ = St_Lower_^0.5 **.**^ St_Higher_^0.5^, ϕ_*r*2Lower_ = ϕ_*r*1_^0.8 **.**^ St_Lower_^0.2^, and ϕ_*r*2Higher_ = ϕ_*r*1_^0.8 **.**^ St_Higher_^0.2^.

Equation 12 predicts that in comparison with results from Experiment 2, QTOE will shift by the amount

13 $$ \Delta \mathrm{QTOE}=\left[\left(1-{s}_1\right)\ \Delta {Q}_1-\left(1-{s}_2\right)\ \Delta {Q}_2\right]\ \left(1/{s}_1+1/{s}_2\right)/2, $$

where Δ*Q*_1_ = (*Q*_1,Exp. 3_ − *Q*_1,Exp. 2)_, and Δ*Q*_2_ = (*Q*_2,Exp. 3 −_*Q*_2,Exp. 2_). From the above, it is predicted that |Δ*Q*_2_| < |Δ*Q*_1_|. This is because ϕ_*r*2_, but not ϕ_*r*1_, is partially adjusted in the direction of the current St duration.

#### Predicting shifts in QTOE

To get an idea of the likely shifts in QTOE between Experiments 2 and 3, rough estimates of *Q*_1_ and *Q*_2_ can be made from the above assumptions, using the NLR results (see Table 3). For Experiment 2, *Q*_1_ and *Q*_2_ are both estimated as −13.6% throughout. For Experiment 3, estimates of *Q*_1_ are +46.7% for St = 100 ms (blocked with 215 ms) and St = 464 ms (blocked with 1,000 ms), and −31.8% for St = 215 ms (blocked with 100 ms) and St = 1,000 ms (blocked with 464 ms); estimates of *Q*_2_ are +35.8% for St = 100 ms and St = 464 ms, and −26.4% for St = 215 ms and St = 1,000 ms. From this we get, for St = 100 ms and 464 ms, Δ*Q*_1_ = +60.3% and Δ*Q*_2_ = +49.4%; and for St = 215 ms and St = 1,000 ms, Δ*Q*_1_ = −18.2% and Δ*Q*_2_ = −12.8%. Also, using the NLR results (see Table 3), *s*_1_ is estimated (for Experiment 2 as well as Experiment 3) as 0.391, 0.526, 0.485, and 0.434 for St = 100; 215; 464; and 1,000 ms, respectively, and *s*_2_ as 0.339, 0.455, 0.536, and 0.729 for the same durations. Using Equation 14, we then roughly predict QTOE shifts of +11.0% (100 ms), −3.4% (215 ms), +15.9 (464 ms), and −12.6% (1,000 ms). Most importantly, these shifts in QTOE are predicted to form a zig-zag pattern when plotted against St duration. This is because as long as *s*_1_ < 1, *s*_2_ < 1, and |Δ*Q*_2_| < |Δ*Q*_1_|, the shift in QTOE will generally be positive in series with St intervals of 100 ms and 464 ms, which are blocked with longer St intervals (215 ms and 1,000 ms, respectively), and negative for series with St intervals of 215 ms and 1,000 ms, which are blocked with shorter St intervals (100 ms and 464 ms, respectively). (A possible exception could occur for [1 − *s*_1_] / [1 − *s*_2_] << 1, for instance, with *s*_1_ close to 1.)

With the standard deviations (*SD*s) of QTOE for Experiment 2 given in Table 7 in the Appendix, the predicted shifts with the four standard durations represent Cohen’s *d* values of 1.15, 0.35, 1.91, and 1.57, respectively. The predicted zig-zag effect (calculated as the mean, 10.75%, of the unsigned shift percentages) represents (as compared with the *SD*, 5.80, of the grand mean QTOE in Experiment 2) a Cohen’s *d* of 1.85, and with the current sample sizes even an effect half as large should be detected with a probability > 0.99 at α = 0.05.

#### Predictions of increased Weber fractions

It was further predicted that, due to the intermixing of St durations in a block, *Q*_1_ and Q_2_ would be less stable across trials in Experiment 3 than in Experiment 2, where the standard was fixed within each block. This would make perception of the duration difference (*d*_12_) in the pair more variable from trial to trial. As a result, WFs would be larger for corresponding conditions in Experiment 3 than in Experiment 2 (cf. Hellström, 2000). The extent of this effect is hard to predict, but a moderate shift, with Cohen’s *d* = 0.5, of the mean WF (across St durations and presentation orders) would be detectable (at α = 0.05) with a power of 0.76.

### Method

#### Participants

Participants were undergraduate psychology students at the University of Bern, 67 females and six males, ranging in age from 18 to 32 years (21.7 ± 2.6 years). The participants received course credit. All of them were naïve about the purpose of the study and reported normal hearing and normal or corrected-to-normal vision. None of them had participated in Experiment 1 or Experiment 2. All participants gave their written informed consent (see Footnote 1).

#### Procedure

Apparatus and stimuli were the same as in Experiment 2. The experimental session comprised a total of eight blocks, with a 1-min break between blocks. In four of the blocks, Co was initially longer than St (Hi-Co blocks) while in the other four blocks Co was initially shorter than St (Lo-Co blocks). Furthermore, the St durations in four of the blocks were short (100 and 215 ms) and in the other four blocks, they were long (464 and 1,000 ms). Each block consisted of two randomly interleaved series of 32 trials each. In one of these series, the stimuli were always presented in the order StCo, and in the other series, in the order CoSt. As in Experiments 1 and 2, series types were StCoU, StCoD, CoStU, and CoStD. If the St duration in the StCo series of a block was 100 (464) ms, the St duration in the CoSt series of the same block was 215 (1,000) ms, and vice versa. Block order was balanced across participants.

### Results

Following Experiments 1 and 2, a Mahalanobis distance criterion of *p* = .025 was applied for outlier detection, which resulted in the exclusion of eight participants, so that analyses are based on *n* = 65.

#### Descriptives

In Table 9, in the Appendix, descriptive statistics for rWF_StCoU_, rWF_StCoD_, rWF_CoStU_, rWF_CoStD_, WF_M_, and QTOE are given for each St duration in Experiment 3, as well as for mean WF_M_ across St durations. Figure 6 shows the mean (*M*) and standard error of the mean (*SEM*) of the WF for each presentation order, as well as the ratio of the estimates of WF_StCo_ and WF_CoSt_ (indicating *s*_1_/*s*_2_).

**Fig. 7:**
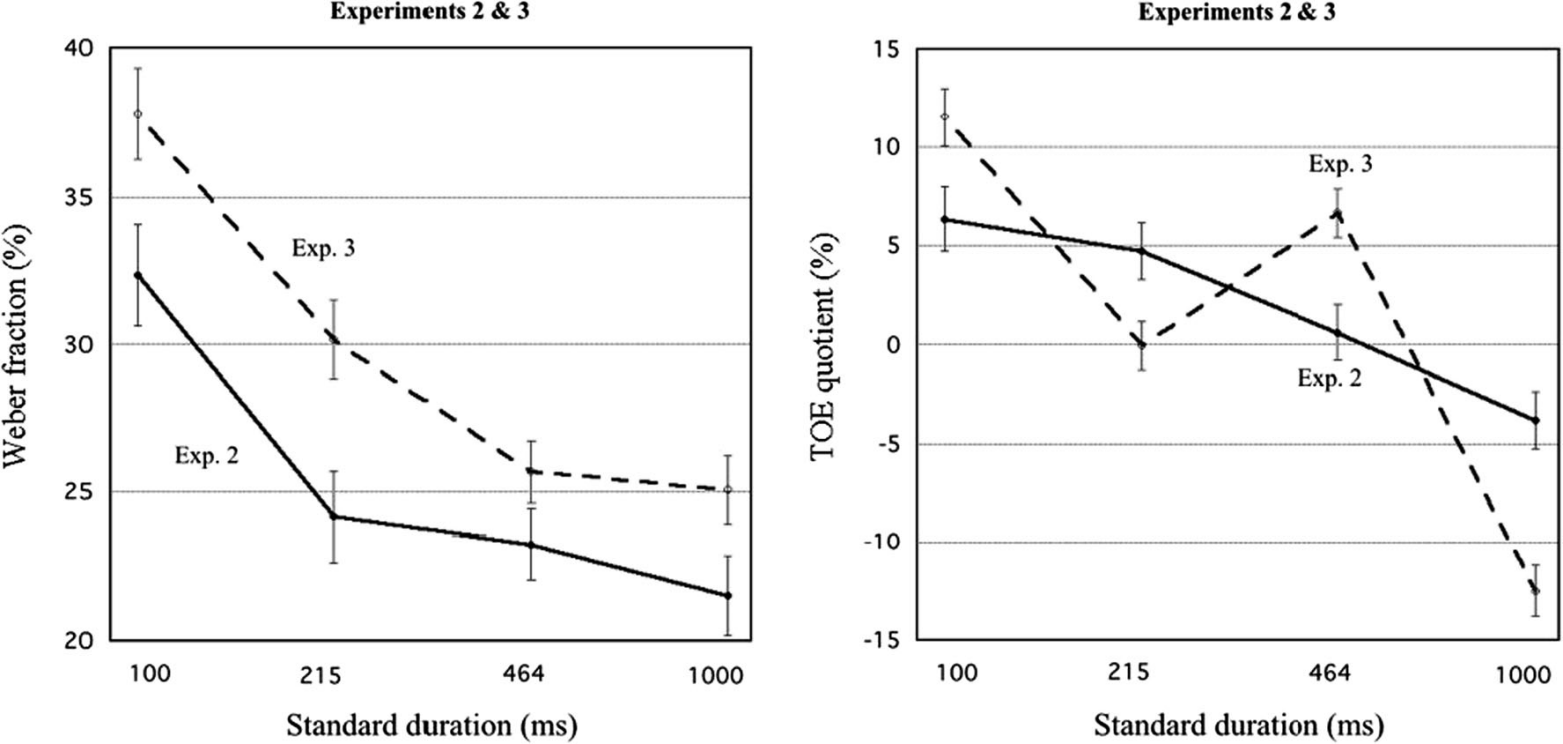
For Experiments 2 and 3 (empty visual intervals), mean Weber fraction across stimulus orders (left) and TOE quotient (QTOE; right) is plotted against standard (St) duration (logarithmic time scale). Error bars indicate the standard error

Figure 7 displays mean WFs (left panel) and QTOEs (right panel) for Experiments 2 and 3 together, plotted against St duration in a logarithmic time scale. As can be seen, WFs show a similar dependence on St duration in Experiment 3 as in Experiment 2, albeit at a higher level. For QTOEs, the results for Experiment 3 depict, when superimposed on the sloping curve from Experiment 2, a zig-zag pattern with maxima for St = 100 ms and St = 464 ms, and minima for St = 215 ms and St = 1,000 ms. The change in QTOE from Experiment 2 to Experiment 3 was, for St = 100 ms, +5.17% (*SEM* = 2.19), for St = 215 ms, −4.80% (*SEM* = 1.91), for St = 464 ms, +6.03% (*SEM* = 1.89), and for St = 1,000 ms, −8.70% (*SEM* = 1.94). The mean change in QTOE in the predicted directions was 6.18% (*SEM* = 0.71).

#### ANOVA results

##### Experiment 3

The WFs and the QTOEs from Experiment 3 were submitted to repeated-measures ANOVAs, with presentation order (StCo, CoSt) and St duration as within-participant factors. The results are given in Table 4.

**Table 4.** ANOVA table for analysis of Weber fractions and QTOEs from Experiment 3 (empty visual intervals)

	Weber fractions				QTOEs			
Effect	*F*	*df*	*p*	η^2^_p_	*F*	*df*	*p*	η^2^_p_
St duration Linear Quadratic Cubic	32.277 82.780 13.635 0.031	3, 62 1, 64 1, 64 1, 64	**< .001** **< .001** **< .001** .862	.610 .564 .176 .000	99.715 199.412 9.569 78.963	3, 62 1, 64 1, 64 1, 64	**< .001** **< .001** **.003** **< .001**	.828 .757 .130 .552
Order	0.975	1, 64	.327	.015	11.201	1, 64	**.001**	.149
Dur. x Order Linear Quadratic Cubic	26.359 67.139 11.793 21.531	3, 62 1, 64 1, 64 1, 64	**< .001** **<.001** **.001** **< .001**	.561 .512 .156 .252	6.285 4.260 12.008 6.271	3, 62 1, 64 1, 64 1, 64	**< .001** **.043** **< .001** **.015**	.233 .062 .158 .089
Type B effect St = 100 ms St = 215 ms St = 464 ms St = 1000 ms	*t* 6.586 -2.796 -2.091 -5.737	*df* 64 64 64 64	*p* **< .001** **.027** .162 **< .001**					

*Note*. Bonferroni-corrected *t*-test results for Weber fraction difference between presentation orders StCo and CoSt (i.e., Type B effect), are also given for each standard duration; *p* values indicating statistical significance (*p* < .05) are given in boldface

##### Weber fractions (WFs)

For WFs, the pattern (see Fig. 6) was similar to that obtained in Experiment 2. Again, the Duration × Order interaction was significant, showing a Type B effect that shifted with St duration. Paired *t* tests (with Bonferroni corrections) of WFs were conducted for orders StCo versus CoSt. For St = 100 ms, another piece of evidence for a positive Type B effect was obtained: WF_StCo_ − WF_CoSt_ > 0, *p* < .001.

##### TOE quotients (QTOEs)

For QTOEs, not only the linear trend of the main effect of duration was statistically significant (*p* < .001) like in Experiment 2, but also the quadratic and cubic trends, confirming the predicted zig-zag pattern. The shifts are smaller than our rough predictions above (which are highly dependent on the estimates of *s*_1_, *s*_2_, *Q*_1_, and *Q*_2_), but what is important is that their zig-zag pattern was correctly predicted. It may well be the case that ReLs are more resilient to manipulation within an experiment (e.g., due to effects of residual stimulation) than we expected.

##### Experiments 2 and 3 together

Each measure (WF, QTOE) was submitted to a repeated-measures ANOVA, with presentation order (StCo, CoSt) and St duration (100; 215; 464; 1,000 ms) as within-participant factors, and experiment (2, 3) as a between-participants factor. The results are shown in Table 5.

**Table 5.** ANOVA tables for analyses of Weber fractions and QTOEs from Experiments 2 and 3 combined

Effect	Weber fractions				QTOEs			
	*F*	*df*	*p*	η_p_^2^	*F*	*df*	*p*	η_p_^2^
St duration	46.904	3, 111	**< .001**	.559	74.820	3, 111	**< .001**	.669
Order	3.161	1, 113	.078	.027	45.909	1, 113	**< .001**	.289
Experiment	8.244	1, 113	**.005**	.068	0.172	1, 113	.679	.002
Dur. × Exp.	1.384	3, 111	.251	.036	24.358	3, 111	**< .001**	.397
Order × Exp.	0.181	1, 113	.672	.002	3.234	1, 113	.075	.028
Dur. × Order	32.007	3, 111	**< .001**	.464	9.322	3, 111	**< .001**	.201
Dur. × Order × Exp.	7.422	3, 111	**< .001**	.167	1.149	3, 111	.333	.030

*Note. p* values indicating statistical significance (*p* < .05) are given in boldface

##### Weber fractions (WFs)

As predicted, WFs were significantly higher in Experiment 3 than in Experiment 2. The *M* (*SD*, *SEM*) of the mean WF was, for Experiment 2, 25.33% (8.19, 1.16) and for Experiment 3, 29.69% (7.98, 0.99), yielding an actual Cohen’s *d* value of 0.54.

##### TOE quotients (QTOEs)

For the QTOEs, the main effects of duration and order were significant, like the Duration × Order interaction. Most importantly, the Duration × Experiment interaction was significant. The effect size, η_p_^2^ = .397, could serve as an index of the degree of impact of the weighting mechanism on the QTOE in the combined Experiments 2 and 3; *p* values were < .001 for the linear and cubic contributions of duration to the interaction, highlighting the contrast of the zig-zag pattern of Experiment 3 with the regular negative slope for Experiment 2 (see Fig. 7, right).

The model used in the analysis of the results from Experiment 3 is not compatible with the simplified model (assuming one single ReL for each St duration), which was used in the multivariate and NLR analyses of data from Experiments 1 and 2. Therefore, no such analyses were conducted on the data from Experiment 3.

#### Response times

Response times in Experiment 3 are reported and discussed in the Appendix.

#### Discussion of Experiment 3

The results of Experiment 3, which are shown in Table 5 and in Fig. 6, confirm the theoretical predictions from the SW model of how QTOEs change as a function of the design-generated level of *Q*_1_. They demonstrate the predictive power of the SW model, and also strengthen the concept of the ReL as the result of pooling of stimulus magnitude information (cf. Helson, 1964). This ReL constitutes a realistic expectation for the duration of the upcoming stimulus interval, which is weighted-in to enhance the efficiency of the comparison process (Patching et al., 2012).

## General discussion

### Type B effects: Not always negative

Ellinghaus et al. (2018) state that “Type B effects reported in the literature . . . are almost exclusively negative . . . . Positive Type B effects have rarely been reported in the case of very short-duration stimuli, especially when presented with very short interstimulus intervals” (p. 8). This may be true for the stimulus conditions usually employed, but this fact seems to be due to researchers’ strange reluctance to use interstimulus intervals other than about 1,000 ms, or stimuli briefer than 500 ms. With shorter interstimulus intervals and/or briefer stimuli, cases of (in terms of the SW model) *s*_1_/*s*_2_ > 1, with large TOEs and positive Type B effects or equivalent results, have been found (Hellström, 1979, 2003; Hellström & Rammsayer, 2004). In our view, to fully explore the effects of stimulus presentation conditions, psychophysical research should not avoid brief stimuli or fast stimulus presentation.

The results of Ellinghaus et al. (2018), which were obtained by using only an interstimulus interval of 1,000 ms and an St duration of 500 ms, across 10 different stimulus types, highlight the similarity between the comparison of durations and of other stimuli. Bausenhart et al. (2015) used auditory durations, with St durations of 100 ms and 1,000 ms, and found consistently negative Type B effects when the interstimulus interval was 1,000 ms. In contrast, when it was 300 ms, there was an interaction of presentation order (StCo, CoSt) and St duration, the Type B effect being negative for St = 1,000 ms, but slightly and nonsignificantly positive for St = 100 ms. Bausenhart et al. (2015) acknowledge that “we cannot refute the findings of a positive Type B effect under specific conditions. . . . A more general framework [than the IR model], such as *Sensation Weighting . . .* would be needed to account for any reversal of the Type B effect” (p. 1038).

The Type B effect can be seen primarily as an indicator of the sensation-weighting balance, but a rather insensitive one, as it is based on the comparison of measures of discrimination, such as DLs. In Experiments 1 and 2, this balance, as evidenced also by the QTOE, was once more found to be heavily dependent on the stimulus conditions. The present results affirm once more (cf. Hellström, 1979, 1985, 2003; Patching et al., 2012) that it is unwarranted to conclude that *s*_1_/*s*_2_< 1 is a general rule in the comparison of successive stimuli.

## Conclusion

Our results demonstrate the necessity of considering, when assessing stimulus discrimination, methodological factors such as the presentation order of St and Co, which are not recognized by the time-honored simple difference model. Even in a design with a single standard duration per stimulus block, TOEs depend systematically on stimulus conditions (here, St duration) in combination with participant-specific factors such as judgment bias and ReL location. This means that a model for comparison of interval durations, and of stimulus magnitudes in general, must be able to account for both the Type B effect and the TOE, as well as for each of these going in either direction. Because it has these capabilities, the SW model has proved useful in previous studies using various study designs and stimulus modalities (e.g., Englund & Hellström, 2012, 2013; Hellström, 1979, 1985, 2000, 2003; Hellström, Aaltonen, Raimo, & Vilkman, 1994; Hellström & Cederström, 2014; Patching et al., 2012). The SW model also predicts the close relation between the TOE and the Type B effect. Although, by necessity, it gives a simplified account of what actually happened in the present experiments, the SW model has once more helped to understand the contributions and the interplay of the perceptual-cognitive factors behind the discrimination and comparison of stimulus magnitudes.

Our multivariate results from Experiments 1 and 2, as well as the univariate results of Experiment 3, provide clear evidence for a reversal of the weighting balance, yielding *s*_1_/*s*_2_ > 1 and thereby positive Type B effects, for brief St durations (cf. Hellström, 1979, 2003; Hellström & Rammsayer, 2004, 2015). This casts doubt on theoretical models, like the MH and IR models, that do not allow for such cases. It is also a serious challenge for such models (e.g., Preuschhof et al., 2010; Raviv et al., 2012) that rest on the notion of Bayesian inference of the true magnitude of the first stimulus from its internal representation, which inevitably yields *s*_1_/*s*_2_ < 1. The limitation of these models seems to be their disregard of the possibility that, for optimality in the *comparison* of the two stimuli, also the true magnitude of the second one has to be inferred. Like the MH and IR models, they consider the representation only of the first stimulus as being subject to modification or supplementation, while the second stimulus enters the comparison in a direct way. Instead, as pointed out by Hellström (1979), both of the stimuli should be seen as being in memory at the time of comparison; an analogy with perceptual aftereffects, affecting the perception of the second out of two successive stimuli, may also be made (cf. Hellström, 1985). In summary, we argue that a more flexible model of stimulus comparison has to be adopted, which allows stimulus weighting to be optimized for this task (Hellström, 1989; Patching et al., 2012). The SW model allows for such weighting, and also suggests an underlying mechanism: the weighting-in of supplementary magnitude information by way of reference levels.

## Appendix

**Table 6:** Summary of the design of Experiments 1 and 2

4 Hi-Co blocks 64 trials per block	4 Lo-Co blocks 64 trials per block
St duration 100 (215; 464; 1,000) ms	St duration 100 (215; 464; 1,000) ms
Initial Co duration 135 (285; 564; 1,500) ms	Initial Co duration 65 (145, 364, 500) ms
Presentation order and series type (Up [U]; Down [D]) StCoU (32 trials) CoStD (32 trials)	Presentation order and series type (Up [U]; Down [D]) StCoD (32 trials) CoStU (32 trials)

*Note.* The four St durations (100; 215; 464; and 1,000 ms) in each block pair were counterbalanced over participants. In each block, each presentation order, StCo and CoSt, was used on 32 randomly interleaved trials (see text for details)

Procedure modification

In our earlier studies (Hellström & Rammsayer, 2004, 2015), the smallest difference in duration between the Co and the St was 1 ms in the direction of its initial value—that is, the Co was not permitted to traverse the duration level of the St and cross over to the opposite side. However, as was found in detailed analyses of the results from Hellström and Rammsayer (2015), this no-crossover rule tends to yield a misrepresentation of results in the presence of a large TOE or bias. For instance, with a large positive TOE in the condition CoStD, Co may have to descend below St in order to reach the upper limen. In the present study the no-crossover rule was therefore removed, so that measured DLs were free to attain nonpositive values.

Here we compare the results of Experiment 1 with those of the analogous Experiment 2 of Hellström and Rammsayer (2015), where the no-crossover rule was in force. Two repeated-measures ANOVAs were conducted, with experiment (Hellström & Rammsayer 2015, present) as a between-participants factor, St duration (100; 215; 464; 1,000 ms) and stimulus presentation order (StCo, CoSt) as within-participant factors, and QTOE and WF, respectively, as the dependent variable. For QTOE, only the effect of St duration reached significance, *F*(3, 113) = 25.170, *p* < .001, η_p_^2^ = .401, but none of the effects involving experiment. For WF, the only significant effects were those of St duration, *F*(3, 113) = 5.856, *p* < .001, η_p_^2^ = .135, and experiment, *F*(1, 115) = 17.414, *p* < .001, η_p_^2^ = .132. WFs tended to be lower in the present Experiment 1 than in Hellström and Rammsayer’s (2015) Experiment 2. The likely reason is that with the no-crossover rule used in the 2015 study, but not in the present one, the individual rDLs could not reach nonpositive values, which might otherwise occur because of strong positive or negative TOEs. For instance, in the present Experiment 1, with the St duration of 100 ms, QTOE was strongly positive (see Fig. 2), and accordingly, in series type CoStD, 18.0% of the rDLs were negative, but only 1.6% in series type CoStU.

### Univariate and multivariate models, as applied to the four series types in Experiments 1 and 2

#### Univariate model

##### Raw DLs

The physical duration of the St is denoted by *S*. For each of the four series types, the left member of each of Equations 14–17 (where *i* [participant] subscripts are omitted)—that is, *w S* or −*w* · *S*, represents the subjective stimulus difference, *d*_12*X*_ or −*d*_12*X*_, which corresponds to a raw DL (rDL_D_ or rDL_U_, respectively). The right member of each equation describes how *w* · *S* or −*w* · *S* is built up in the particular series type:

14 $$ \mathrm{StCoU}:-w\cdotp S={s}_1\cdotp S-{s}_2\ \left(S+{\mathrm{rDL}}_{\mathrm{StCoU}}\right)+\left({s}_2-{s}_1\right)\ {\upphi}_r+b\cdotp S; $$

15 $$ \mathrm{StCoD}:w\cdotp S={s}_1\cdotp S-{s}_2\ \left(S-{\mathrm{rDL}}_{\mathrm{StCoD}}\right)+\left({s}_2-{s}_1\right)\ {\upphi}_r+b\cdotp S; $$

16 $$ \mathrm{CoStU}:-w\cdotp S={s}_1\ \left(S-{\mathrm{rDL}}_{\mathrm{CoStU}}\right)-{s}_2\cdotp S+\left({s}_2-{\mathrm{s}}_1\right)\ {\upphi}_r+b\cdotp S; $$

17 $$ \mathrm{CoStD}:w\cdotp S={s}_1\ \left(S+{\mathrm{rDL}}_{\mathrm{CoStD}}\right)-{s}_2\cdotp S+\left({s}_2-{s}_1\right)\ {\upphi}_r+b\cdotp S. $$

##### Raw Weber fractions

For comparability of effects between the four St durations, each rDL was transformed into a raw Weber fraction (rWF): rWF = rDL/*S*. Expressions are obtained from Equations 14–17 that describe how the rWF is built up, according to the SW model, in each series type:

18 $$ \mathrm{StCoU}:{\mathrm{rWF}}_{\mathrm{StCoU}}=\left(1/{s}_2\right)\ \left[w+b+\left({s}_2-{s}_1\right)\ Q\right]; $$

19 $$ \mathrm{StCoD}:{\mathrm{rWF}}_{\mathrm{StCoD}}=\left(1/{s}_2\right)\ \left[w-b-\left({s}_2-{s}_1\right)\ Q\right]; $$

20 $$ \mathrm{CoStU}:{\mathrm{rWF}}_{\mathrm{CoStU}}=\left(1/{s}_1\right)\ \left[w+b+\left({s}_2-{s}_1\right)\ Q\right]; $$

21 $$ \mathrm{CoStD}:{\mathrm{rWF}}_{\mathrm{CoStD}}=\left(1/{s}_1\right)\ \left[w-b-\left({s}_2-{s}_1\right)\ Q\right], $$

where *Q* is the ReL distance quotient, that is, the relative distance of the ReL from the St: *Q* = (ϕ_*r*_ − *S*)/*S*. (Unlike in Hellström and Rammsayer, 2015, a logarithmic transformation of the rWFs could not be used, as individual rWF values were free to be nonpositive, as explained above. Also, the present approach differs from that used in the 2015 article in that the possible difference in ϕ_*r*_ between Hi-Co and Lo-Co blocks is ignored.)

**Table 7:** Experiments 1 and 2: Descriptive statistics of raw Weber fraction (rWF, in %), Weber fraction (WF, in %), and TOE quotient (QTOE, in %) for each experiment

	Experiment 1: Filled auditory intervals (*N* = 61)			Experiment 2: Empty visual intervals (*N* = 50)		
	*M*	*SEM*	*SD*	*M*	*SEM*	*SD*
St = 100 ms						
rWF_StCoU_	18.77	1.39	10.89	41.79	2.32	16.43
rWF_StCoD_	9.31	0.85	6.65	26.48	2.13	15.06
rWF_CoStU_	18.61	1.19	9.28	35.64	2.32	16.40
rWF_CoStD_	8.86	1.38	10.77	25.50	2.95	20.89
WF_StCo_	14.04	0.88	6.85	34.14	1.81	12.77
WF_CoSt_	13.73	0.93	7.25	30.57	1.89	13.38
WF_M_	13.89	0.81	6.32	32.35	1.64	11.61
QTOE	+4.80	0.70	5.49	+6.36	1.36	9.59
St = 215 ms						
rWF_StCoU_	15.47	1.25	9.74	34.86	2.72	19.20
rWF_StCoD_	8.49	0.93	7.28	14.66	1.85	13.09
rWF_CoStU_	14.94	1.17	9.17	22.98	1.75	12.36
rWF_CoStD_	8.28	1.03	8.05	24.19	2.59	18.32
WF_StCo_	11.98	0.81	6.29	24.76	1.72	12.13
WF_CoSt_	11.61	0.67	5.24	23.59	1.54	10.90
WF_M_	11.79	0.59	4.59	24.17	1.42	10.01
QTOE	+3.41	0.68	5.34	+4.75	1.39	9.86
St = 464 ms						
rWF_StCoU_	13.24	0.96	7.46	26.16	2.58	18.22
rWF_StCoD_	7.95	0.94	7.36	20.19	1.48	10.46
rWF_CoStU_	14.19	0.95	7.41	21.59	1.88	13.32
rWF_CoStD_	9.31	1.02	7.95	25.05	2.00	14.11
WF_StCo_	10.60	0.64	5.01	23.18	1.62	11.46
WF_CoSt_	11.75	0.65	5.11	23.32	1.36	9.59
WF_M_	11.17	0.52	4.07	23.25	1.27	8.99
QTOE	+2.54	0.66	5.16	+0.63	1.18	8.36
St = 1,000 ms						
rWF_StCoU_	10.41	1.27	9.92	15.45	2.63	18.62
rWF_StCoD_	12.40	1.20	9.37	16.55	2.36	16.66
rWF_CoStU_	10.35	1.41	10.99	20.02	1.81	12.82
rWF_CoStD_	17.35	1.78	13.87	34.16	2.28	16.11
WF_StCo_	11.41	0.77	6.03	16.00	1.93	13.65
WF_CoSt_	13.85	1.01	7.89	27.09	1.45	10.26
WF_M_	12.63	0.78	6.12	21.545	1.38	9.78
QTOE	-2.25	1.00	7.80	-3.81	1.14	8.06
Mean WF_M_	12.37	0.49	3.83	25.33	1.16	8.19

### Multivariate model

In the multivariate analysis, we attempt to describe each measured rWF as a sum of products of participant-specific and condition-specific factors. In our multivariate model, then, the *i*th *participant* is characterized by this participant’s (1) Weber constant, *w*_*i*_; (2) judgment bias, *b*_i_, and (3) ReL distance quotient, *Q*_*i*_. The *k*th stimulus condition (defined by St duration and series type—StCoU, StCoD, CoStU, or CoStD) is characterized by this condition’s (1) discrimination difficulty, ω_*k*_, (2) sensitivity to bias, β_*k*_ or −β_*k*_, and (3) weight difference, δ_*k*_ (i.e., [*s*_2*k*_ − *s*_1*k*_] or −[*s*_2*k*_ − *s*_1*k*_])—that is, the multiplier of *Q*_*i*_ in determining the TOE. (As the analysis was based on correlations, not on covariances, all variables are considered standardized, and the factors 1/*s*_1_ and 1/*s*_2_, which can be expected to vary across St durations, are left outside the analysis.) Thus, the predicted rWF for the *i*th participant in the *k*th condition is:

22 $$ {\mathrm{rWF}}_{\mathrm{ik}}={\upomega}_k\ {w}_i+{\upbeta}_k\ {b}_i+{\updelta}_k\ {Q}_i. $$

The last term is built on the assumption that the ReL distance quotient (*Q*) is participant specific and (comparatively) invariant across stimulus conditions, while weighting (i.e., *s*_2_ − *s*_1_) is condition specific and (comparatively) invariant across participants. This assumption we consider sufficiently justified by the univariate results, which indicate that *s*_2_ − *s*_1_ changes with the standard duration, whereas *Q* stays negative across conditions.

**Table 8:** Experiments 1 and 2: Unrotated component loadings from principal component analyses of correlations between raw Weber fractions (rWFs). Estimated mean values of model parameters ω, β, and δ (Equation 22) for each St duration

	Experiment 1: Filled auditory intervals			Experiment 2: Empty visual intervals		
Component No. Interpretation Eigenvalue	2 *w* 3.1	1_rev_ *b* 3.9	3 *Q* 1.6	1 *w* 4.4	2_rev_ *b* 2.8	3 *Q* 1.7
St = 100 ms						
rWF_StCoU_	.656	.260	−.261	.623	.271	.269
rWF_StCoD_	.534	−.388	.293	.504	−.439	.049
rWF_CoStU_	.512	.269	−.446	.616	.251	−.422
rWF_CoStD_	.425	−.446	.262	.436	−.549	.258
Mean ω, β*,* δ	.532	.340	−.315	.545	.378	−.249
St = 215 ms						
rWF_StCoU_	.415	.518	−.180	.673	.334	−.209
rWF_StCoD_	.558	−.220	.309	.327	−.520	−.043
rWF_CoStU_	.430	.348	−.507	.633	.433	−.165
rWF_CoStD_	−.019	−.552	.307	.290	−.778	.023
Mean ω, β*,* δ	.346	.410	−.326	.481	.516	−.089
St = 464 ms						
rWF_StCoU_	.506	.424	.217	.641	.339	.403
rWF_StCoD_	.302	−.719	−.113	.364	−.468	−.004
rWF_CoStU_	.447	.653	.083	.628	.274	.393
rWF_CoStD_	.432	−.602	−.179	.380	−.537	−.484
Mean ω, β*,* δ	.422	.599	.148	.503	.404	.321
St = 1,000 ms						
rWF_StCoU_	.153	.609	.464	.547	.154	.380
rWF_StCoD_	.396	−.544	.129	.517	.387	−.169
rWF_CoStU_	.352	.483	.553	.450	−.228	.637
rWF_CoStD_	.454	−.531	−.208	.490	−.333	−.375
Mean ω, β*,* δ	.339	.542	.234	.501	−.032	.390

*Note.*_rev_ loading signs are reversed

### Experiment 3: Manipulation of ReLs

With the two ReLs potentially different, we may rewrite Equation 3 as

23 $$ {d}_{12}={\upphi}_1-{\upphi}_2+\left(1-{s}_1\right)\ \left({\upphi}_{r1}-{\upphi}_1\right)-\left(1-{s}_2\right)\ \left({\upphi}_{r2}-{\upphi}_2\right)+b, $$

from which, setting *d*_12_ = *d*_12*X*_ = *w* · *S*, we obtain the following equations for rWF in the four series types (presentation orders StCo and CoSt; Up [U] and Down [D]):

24 $$ \mathrm{StCoU}:{\mathrm{rWF}}_{\mathrm{StCoU}=}\ \left[w+\left(1-{s}_1\right)\ {Q}_1-\left(1-{s}_2\right)\ {Q}_2+b\right]/{s}_2; $$

25 $$ \mathrm{StCoD}:{\mathrm{rWF}}_{\mathrm{StCoD}}=\left[w-\left(1-{s}_1\right)\ {Q}_1+\left(1-{s}_2\right)\ {Q}_2-b\right]/{s}_2; $$

26 $$ \mathrm{CoStU}:{\mathrm{rWF}}_{\mathrm{CoStU}}=\left[w+\left(1-{s}_1\right)\ {Q}_1-\left(1-{s}_2\right)\ {Q}_2+b\right]/{s}_1; $$

27 $$ \mathrm{CoStD}:{\mathrm{rWF}}_{\mathrm{CoStD}}=\left[w-\left(1-{s}_1\right)\ {Q}_1+\left(1-{s}_2\right)\ {Q}_2-b\right]/{s}_1, $$

where *Q*_1_ = (ϕ_*r*1_ − *S*)/*S* and *Q*_2_ = (ϕ_*r*2_ − *S*)/*S*.

**Table 9:** Experiment 3: Descriptive statistics of raw Weber fraction (rWF, in %), Weber fraction (WF, in %), and TOE quotient (QTOE, in %)

	Experiment 3:Empty visual intervals (*N* = 65)		
	*M*	*SEM*	*SD*
St = 100 ms			
rWF_StCoU_	55.19	2.83	22.79
rWF_StCoD_	35.78	3.27	26.38
rWF_CoStU_	43.50	2.72	18.32
rWF_CoStD_	16.78	2.65	21.38
WF_StCo_	45.48	2.15	17.36
WF_CoSt_	30.14	1.70	13.70
WF_M_	37.81	1.55	12.50
QTOE	+11.54	1.61	12.96
St = 215 ms			
rWF_StCoU_	31.07	2.10	16.93
rWF_StCoD_	22.64	1.51	12.21
rWF_CoStU_	29.15	3.36	27.07
rWF_CoStD_	37.80	2.63	21.18
WF_StCo_	26.86	1.26	10.18
WF_CoSt_	33.48	2.26	18.24
WF_M_	30.17	1.40	11.27
QTOE	−0.06	1.28	10.35
St = 464 ms			
rWF_StCoU_	33.45	2.30	18.55
rWF_StCoD_	15.36	1.65	13.30
rWF_CoStU_	31.27	1.57	12.65
rWF_CoStD_	22.71	1.96	15.79
WF_StCo_	24.40	1.24	9.97
WF_CoSt_	26.99	1.12	9.00
WF_M_	25.70	1.00	8.08
QTOE	+6.66	1.33	10.75
St = 1,000 ms			
rWF_StCoU_	9.80	1.76	14.15
rWF_StCoD_	30.23	2.30	18.54
rWF_CoStU_	15.34	2.07	16.67
rWF_CoStD_	44.94	2.85	23.01
WF_StCo_	20.01	1.28	10.31
WF_CoSt_	30.14	1.60	12.88
WF_M_	25.08	1.15	9.24
QTOE	−12.51	1.46	11.76
Mean WF_M_	29.69	0.99	7.98

### Response times (RTs)

The arithmetic mean RT (MRT) was calculated across the last 20 trials of each series. Descriptive statistics are given in Table 10. For each experiment, the MRTs were submitted to a repeated-measures ANOVA, with St duration presentation order, and series profile (U, D) as within-participant factors. For Experiment 1, statistically significant effects were found for duration, *F*(3, 58) = 8.294, *p* < .001, η_p_^2^ = .300, and for profile, *F*(1, 60) = 118.045, *p* < .001, η_p_^2^ = .663. For Experiment 2, significant effects were found for profile, *F*(1, 49) = 33.540, *p* < .001, η_p_^2^ = .406, and for the Order × Profile interaction, *F*(1, 49) = 5.165, *p* = .027, η_p_^2^ = .095. For Experiment 3, statistically significant effects were found for order, *F*(1, 64) = 9.987, *p* = .002, η_p_^2^ = .135; profile, *F*(1, 64) = 37.115, *p* < .001, η_p_^2^ = .367; and the Duration × Profile interaction, *F*(3, 62) = 10.629, *p* < .001, η_p_^2^ = .340.

As is seen from Table 10, MRTs consistently tended to be longer for D series (with 75% “first longer” responses) than for U series (with 75% “second longer” responses). One possible explanation for this general tendency might be that in some U trials, the participant decided to respond “second longer” before the second duration was ended. However, only for the 1,000-ms standard could this possibly account for the MRT difference, which is on the order of 100 ms for all St durations in Experiments 1 and 2. Another explanation might be that the “second longer” key could be reached somewhat faster than the “first longer” one. However, this is unlikely to yield such a large effect (see also Footnote 2). Also, it would suggest an explanation of the observed judgment bias in terms of fast, careless responses. However, this would predict an excess of “second longer” over “first longer” responses—that is, a negative bias, not a positive one, as was observed. Also, instructions emphasized accuracy, not speed.

More plausibly, the positive judgment bias is due to a tendency to respond “first longer” when the subjective duration difference is too small to categorize (García-Pérez & Alcalá-Quintana, 2019). The MRT difference can then be seen as a sequel of the bias in conjunction with the employed adaptive method: in D series, a positive bias will automatically lead to the presentation of stimulus pairs with less positive (closer to zero) differences between the first and second durations in order not to exceed 75% “first longer” responses. Also, in U series, a positive bias leads to the presentation of stimulus pairs with more negative (farther from zero) differences in order to reach 75% “second longer” responses. As choice RTs tend to be longer for smaller stimulus differences (Link, 1992; Patching et al., 2012), this effect tends to lengthen the majority responses (“first longer”) in D series, and to shorten the majority responses (“second longer”) in U series. The effect is modulated by the occurrence of perceptual TOEs, which shift the point of subjective equality and may thereby lead to interactions of, for instance, Profile × Order, as was found in Experiment 2.

The mean MRT difference between U and D series was computed for each participant, and its correlations with the component scores from the two PCAs were analyzed. The only correlations of the U–D MRT difference that reached or approached significance were those with the component identified with the bias term *b*. For Experiment 1, these correlations were −.286, *p* = .025, with (the reversed) Component 1; for Experiment 2, they were −.343, *p* = .015, with (the reversed) Component 2. These correlations confirm that a more positive *b* value (i.e., a greater tendency to judge “first longer”) is associated with a shorter MRT for U series than for D series, and thereby strengthen the above account of the U–D difference in MRT as a consequence of how the positive judgment bias dictates the allocation of presented stimuli in the employed adaptive staircase method.

**Table 10.** Experiments 1 and 2: Descriptive statistics (mean, standard error of mean) for mean response time (MRT) in milliseconds (ms) in each condition of each experiment

	Experiment 1:Filled auditory intervals (*N* = 61)		Experiment 2:Empty visual intervals (*N* = 50)		Experiment 3:Empty visual intervals (*N* = 65)	
	*M*	*SEM*	*M*	*SEM*	*M*	*SEM*
St =100 ms						
StCoU	1,039	50	1,071	50	1,143	55
StCoD	1,168	56	1,249	68	1,216	67
CoStU	1,057	46	1,138	56	1,233	56
CoStD	1,207	58	1,162	52	1,225	57
StCo	1,104	50	1,160	53	1,180	52
CoSt	1,132	50	1,150	51	1,229	51
U	1,048	45	1,105	49	1,188	50
D	1,187.5	55	1,205	56	1,220	55
Mean	1,118	49	1,155	51	1,204	50
St =215 ms						
StCoU	1,015	56	1,043	51	1,145	55
StCoD	1,202	61	1,136	59	1,163	49
CoStU	1,029	56	1,087	61	1,253	71
CoStD	1,140	62	1,149	62	1,205	66
StCo	1,109	55	1,090	52	1,154	52
CoSt	1,085	56	1,118	58	1,229	60
U	1,022	52	1,065	54	1,199	60
D	1,171	58	1,142	57	1,184	54
Mean	1,097	54	1,104	54	1,191	54
St =464 ms						
StCoU	939	43	1,084	64	1,098	45
StCoD	1,066	44	1,231	69	1,267	58
CoStU	986	48	1,059	50	1,149	51
CoStD	1,021	39	1,149	49	1,265	58
StCo	1,002	38	1,158	62	1,182	47
CoSt	1,003	41	1,104	46	1,207	48
U	963	42	1,072	51	1,123	42
D	1,043	37	1,190	55	1,266	53
Mean	1,003	38	1,131	52	1,195	45
St =1,000 ms						
StCoU	932	55	1,106	59	1,093	56
StCoD	1,011	53	1,273	68	1,251	56
CoStU	898	42	1,129	52	1,081	58
CoStD	981	47	1,246	56	1,254	51
StCo	972	49	1,190	57	1,172	51
CoSt	940	43	1,188	50	1,168	51
U	915	46	1,118	49	1,087	52
D	996	47	1,260	58	1,252	50
Mean	956	45	1189	52	1,170	49
All St durations						
StCo	1,047	41	1149	46	1,172	47
CoSt	1,040	41	1140	44	1,208	48
U	987	40	1090	42	1,149	46
D	1,100	43	1199	48	1,231	50
Grand mean	1,043	41	1145	44	1,190	47

**Table 11:** Abbreviations used in the present article

Abbreviation	Meaning
*b*	Judgment bias
*C*	Physical magnitude of comparison stimulus (Co)
Co	Comparison stimulus varied in physical magnitude (i.e., duration) from trial to trial
CoSt	Stimulus presentation order comparison (Co) followed by standard (St)
D	Series type Down: Second stimulus interval initially shorter than the first
*d*_12_	Subjective difference between the first and the second stimulus
*d*_12*X*_	Subjective difference between the first and the second stimulus at the measured limen in Down (D)-series
*−d*_12*X*_	Subjective difference between the first and the second stimulus at the measured limen in Up (U)-series
DL	Difference limen: half the difference between the upper limen (value of Co that evokes 75% judgments of Co > St) and the lower limen (value of Co that evokes 75% judgments of Co < St)
IR model	Internal reference model
MH model	Michels–Helson model
ϕ	Phi: physical stimulus magnitude; used to represent subjective stimulus and ReL magnitude, assuming the psychophysical function to be the identity function
ψ_1_, ψ_2_	Subjective magnitude of the first stimulus and second stimulus, respectively
ψ	Psi: Subjective magnitude of the stimuli when ψ_1_ = ψ_2_
ψ_*r*1,_ ψ_*r*2_	Magnitude of reference levels (ReL_1_, ReL_2_) for ψ_1_ and ψ_2_ respectively
ψ_*r*_	Magnitude of reference level (ReL) when ψ_*r*1_ = ψ_*r*2_
*Q*	Relative distance of ReL from the St: *Q* = (ϕ_*r*_ − *S*)/*S*
*Q*_1_	Relative distance of first stimulus’ ReL from the St: *Q* = (ϕ_*r*1_ − *S*)/*S*
*Q*_2_	Relative distance of second stimulus’ ReL from the St: *Q* = (ϕ_*r*2_ − *S*)/*S*
QTBE	Type B effect quotient: Type B effect as a fraction of WF_M_
QTOE	TOE quotient: Time order error (TOE) as a fraction of *S*
rDL	Raw difference limen: Difference limen estimated on the basis of the average physical duration of the Co over the last 20 trials of the staircase procedure for each experimental condition
ReL	Reference level
rWF	Raw Weber fraction: Estimated by dividing the raw difference limen (rDL) by the physical duration of the standard (St) stimulus, rDL / St
*S*	Physical magnitude (i.e., duration) of St
*s*_1_, *s*_2_	Weighting coefficients of ψ_1_ and ψ_2_, respectively
St	Standard stimulus held at constant physical magnitude (i.e., duration) in each experimental condition
StCo	Stimulus presentation order standard (St) followed by comparison (Co)
SW model	Sensation weighting model
TBE	Type B effect: Difference between Weber fractions (in %) for presentation orders StCo and CoSt: TBE = WF_StCo −_ WF_CoSt_. Also called standard position effect.
TOE	Time order error (Fechner, 1860): systematic underestimation or overestimation of one stimulus relative to the other (also called Type A effect).
U	Series type Up: Second stimulus interval initially longer than the first
*w*_*i*_	Participant-specific value of the Weber constant, that is, the comparatal dispersion, σ_*d*12_ (after Gulliksen, 1958), multiplied by 0.6745 (i.e., the standard normal deviate corresponding to the 75th percentile) and divided by standard duration *S*. The subscript *i* is dropped in subsequent renditions.
WF	Weber fraction: Traditionally defined as ΔI / I, where ΔI is the difference threshold and I the standard stimulus intensity. Here, estimated by dividing the measured difference limen by the physical duration of the standard, DL / *S*, and expressed in percentage.
WF_M_	Mean WF across stimulus presentation orders (StCo, CoSt).

## Footnotes

1 The study was approved by the ethics committee of the Faculty of Human Sciences of the University of Bern, Bern, Switzerland (date of approval: September 27, 2016; project identification code: 2016-9-00005).

2 These keys were “+” and “Enter,” respectively, which were located on the extreme right-hand side of the keyboard, with the “+” key located above the “Enter” key. Earlier pilot studies showed no evidence for any effect of response key designation on response times.

3 According to Kaiser (1974) KMO values of >.5 are acceptable, and values of .6–.7 are “mediocre.”
